# A hemocyte gene expression signature correlated with predictive capacity of oysters to survive *Vibrio* infections

**DOI:** 10.1186/1471-2164-13-252

**Published:** 2012-06-18

**Authors:** Rafael Diego Rosa, Julien de Lorgeril, Patrick Tailliez, Roman Bruno, David Piquemal, Evelyne Bachère

**Affiliations:** 1 1Ifremer, CNRS, Université Montpellier 2, IRD and Université Montpellier 1, UMR 5119 “Laboratoire Écologie des Systèmes Marins Côtiers, Place Eugène Bataillon, PO Box 34095, Montpellier, France; 2 Université Montpellier 2, IRD,Ifremer, CNRS, and Université Montpellier 1, UMR 5119 “Laboratoire Écologie des Systèmes Marins Côtiers, Place Eugène Bataillon, PO Box 34095, Montpellier, France; 3 INRA, Université Montpellier 2, UMR 1133 “Laboratoire Écologie Microbienne des Insectes et Interactions Hôte-Pathogènes, Place Eugène Bataillon, PO Box 34095, Montpellier, France; 4 Skuldtech, Cap Delta, PO Box 34790, Grabels, France

**Keywords:** Marine invertebrate, Mollusk bivalve, Mass mortality, Transcriptome-wide analysis, Digital gene expression, Microfluidic qPCR, Survival signature, Polymorphism, Gene copy number, Survival predictive biomarkers

## Abstract

**Background:**

The complex balance between environmental and host factors is an important determinant of susceptibility to infection. Disturbances of this equilibrium may result in multifactorial diseases as illustrated by the summer mortality syndrome, a worldwide and complex phenomenon that affects the oysters, *Crassostrea gigas.* The summer mortality syndrome reveals a physiological intolerance making this oyster species susceptible to diseases. Exploration of genetic basis governing the oyster resistance or susceptibility to infections is thus a major goal for understanding field mortality events. In this context, we used high-throughput genomic approaches to identify genetic traits that may characterize inherent survival capacities in *C. gigas*.

**Results:**

Using digital gene expression (DGE), we analyzed the transcriptomes of hemocytes (immunocompetent cells) of oysters able or not able to survive infections by *Vibrio* species shown to be involved in summer mortalities. Hemocytes were nonlethally collected from oysters before *Vibrio* experimental infection, and two DGE libraries were generated from individuals that survived or did not survive. Exploration of DGE data and microfluidic qPCR analyses at individual level showed an extraordinary polymorphism in gene expressions, but also a set of hemocyte-expressed genes whose basal mRNA levels discriminate oyster capacity to survive infections by the pathogenic *V. splendidus* LGP32. Finally, we identified a signature of 14 genes that predicted oyster survival capacity. Their expressions are likely driven by distinct transcriptional regulation processes associated or not associated to gene copy number variation (CNV).

**Conclusions:**

We provide here for the first time in oyster a gene expression survival signature that represents a useful tool for understanding mortality events and for assessing genetic traits of interest for disease resistance selection programs.

## Background

Since the mid-1970s, large-scale episodic events such as harmful algal blooms and other microbial outbreaks, disease epidemics and mass mortalities have occurred in marine environments at a historically unprecedented rate, and may reflect environmental changes and an unfavorable energetic balance in animals [1]. Anthropogenic global changes (climate warming, pollution, introduced species) have been implicated as contributors to disease outbreaks in marine ecosystems worldwide. One aspect of these changes is shifts in natural systems, altering inter-organism dynamics including many host-pathogen interactions and leading to the establishment of new diseases or increased pathogenicity and virulence of established diseases [2]. These emerging infectious diseases have been recently recognized as a threat, not only to humans, but also to global biodiversity [3].

It is widely established that the susceptibility of organisms to infection is determined by a complex balance of environmental and host factors. Disturbances of this equilibrium may result in multifactorial diseases as currently shown in marine invertebrates [4]. Pacific oysters Crassostrea gigas have repeatedly suffered abnormal mortalities during the summer months around the world. Collectively, these mortality events are called summer mortality syndrome, a phenomenon that has been suggested to have a multifactorial etiology, resulting from the combination of multiple biotic and abiotic factors. Those factors include the environment (trophic conditions, elevated water temperature), aquaculture practices, oyster genetics and physiology, reproductive effort followed by spawning and the presence of opportunistic microorganisms [5]. Since 2008, an herpesvirus, OsHV-1 (for Ostreid herpesvirus type 1) [6,7], and various *Vibrio* species [8] have been systematically associated with these abnormal mortalities.

Understanding the role of genetic basis in oyster resistance or susceptibility to infection should deeply contribute to identify the causes of mortalities and the traits that characterize enhanced survival capacities in oyster populations. In recent years, high-throughput genomic approaches have greatly improved knowledge on the genetic basis governing the mechanisms of resistance to infectious diseases, a major challenge for the modern human and veterinary medicine. Several studies have supported a significant role for host genetics in differential susceptibility to many human infectious diseases such as tuberculosis, leprosy, hepatitis, malaria and also AIDS [9,10]. Genome-wide studies have evidenced genomic polymorphic regions (Single Nucleotide Polymorphism or SNP) correlated to changes in the susceptibility to some infectious agents [11]. In the recent years, genomic features such as gene copy number variations (CNV) and transcriptional signatures [12,13] have been extensively investigated as contributing to disease susceptibility or resistance phenotypes. Transcriptional (or gene expression) signatures comprise a combination of genes whose expression patterns are markers of a physiological status [12,14]. In human medicine, gene expression signatures have substantially aided in the diagnosis of cancer and the risk of developing active tuberculosis [12,14-16].

Here, we have investigated potential genetic basis for *C. gigas* resistance to *Vibrio* infections by high-throughput transcriptomic and genomic approaches. For this non model species, the whole genome sequence is still not published [17] but 29,745 unique EST sequences are publicly available (http://public-contigbrowser.sigenae.org:9090/Crassostrea_gigas/index.html) [18]. Thus, we applied the DGE (Digital Gene Expression) deep-sequencing technology. Similar to the massively parallel signature sequencing (MPSS) approach [19], the DGE tag-based approach provides large-scale data sets to establish a complete quantitative and qualitative analysis of cell transcriptome without prior knowledge of genome annotation [20]. In a previous study, the DGE technology was successfully applied for the characterization of defense mechanisms related to the oyster successful response and survival to virulent *Vibrio* infections [21]. Here, the aim was to identify a combination of genes whose inherent basal expression can predict the oyster capacity to survive infections. For that, the hemocytes (immunocompetent cells) have been collected from oysters before an infection by virulent *Vibrio* strains isolated during summer mortality episodes [8]*.* Further monitoring of mortalities allowed classifying samples with contrasted phenotypes of survival or non survival capacity that have been used for the construction of DGE libraries devoted to a predictive character of oysters to circumvent *Vibrio* infection. Through the exploration of DGE data and individual gene expression study by microfluidic qPCR devices [22], we have evidenced a 14-gene survival signature. Besides, we show here for the first time an extraordinary individual polymorphism of hemocyte basal gene expression in oysters.

## Results

### Hemocyte transcriptome of oysters with an inherent capacity to survive *Vibrio* infections

A nonlethal sampling method for hemolymph collection [23] was applied to obtain the hemocyte basal gene expression profile before a challenge by pathogenic bacteria. Hemocyte samples were collected from 180 tagged oysters that were further experimentally infected after an 8-day recovery. *Vibrio* strains, *V. splendidus* LGP32 (8 x 10^7^ CFU/oyster) or *V. aestuarianus* LPi 02/41 (2 x 10^7^ CFU/oyster), which were isolated during 2006 summer mortality episodes in France, were used for experimental infections. Survival rates were 59 % and 33 % (at day 4) for animals infected by *V. splendidus* LGP32 and *V. aestuarianus* LPi 02/41, respectively. Hemocyte mRNA samples collected prior to *Vibrio* infection were then categorized according to oyster survival. Thus, two DGE libraries were constructed from hemocytes of oysters able or not able to survive *Vibrio* infections. They were named SVir-8d [GEO: GSM667902] and NSVir-8d [GEO: GSM667901], respectively.

**Figure 1:**
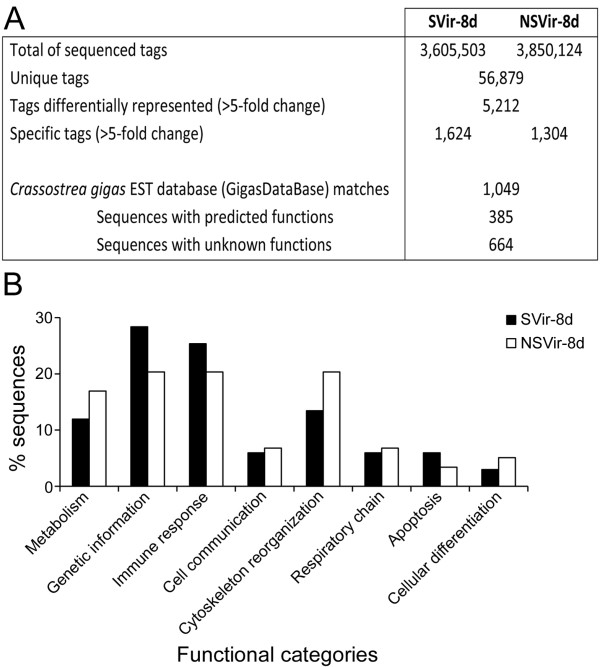
**Hemocyte transcriptome of oysters with distinct capacities to survive *Vibrio *infections.** (**A**) General characteristics of DGE libraries generated from oysters able (SVir-8d) and not able (NSVir-8d) to survive infections by the pathogens *Vibrio splendidus* LGP32 and *V. aestuarianus* LPi 02/41. (**B**) Functional annotation of EST-matched sequences differentially represented between DGE libraries (>5-fold change).

A total of 3,605,503 and 3,850,124 tag sequences (17-bp) were obtained from the whole sequencing of SVir-8d and NSVir-8d libraries, respectively, corresponding to 56,879 unique tags. A comparison of tag occurrences (incidence of each tag in libraries) revealed that 5,212 unique tags were differentially represented between libraries (>5-fold change) (Figure 1A). Tag-mapping was performed against the *C. gigas* EST database (29,745 unique sequences: 7,940 contigs and 21,805 singletons) containing 13,898 ESTs from hemocytes [18]. From the 1,049 EST-matched sequences, only 385 sequences showed homologies to known proteins. Thus, no striking relationship between biological functions and oyster capacity to survive infections was clearly evidenced. However, the SVir-8d library appears to be more enriched in genes related to genetic information processing and immune response compared to NSVir-8d, which was enriched in metabolism and cytoskeleton reorganization (Figure 1B).

### Oyster hemocyte transcriptome-wide analysis reveals a high polymorphism in basal gene expression at individual level

To assess whether gene expression profiles were related to survival capacity phenotypes, we have explored the DGE data on individual oysters by high-throughput microfluidic qPCR approach [22]. Three new independent experimental infections were conducted to obtain individual hemocyte samples from oysters able (S) and not able (NS) to survive infection, as previously done for DGE library construction. We focused on *V. splendidus* which has been shown dominant in field mortalities, since 2009, comparatively to *V. aestuarianus*. Final survival rates of 50 % were obtained and, among the 180 hemolymph samples issued from the three independent infections, 90 samples, corresponding to 45 oysters for each phenotype, yielded high quality RNA suitable for further analyses.

**Figure 2:**
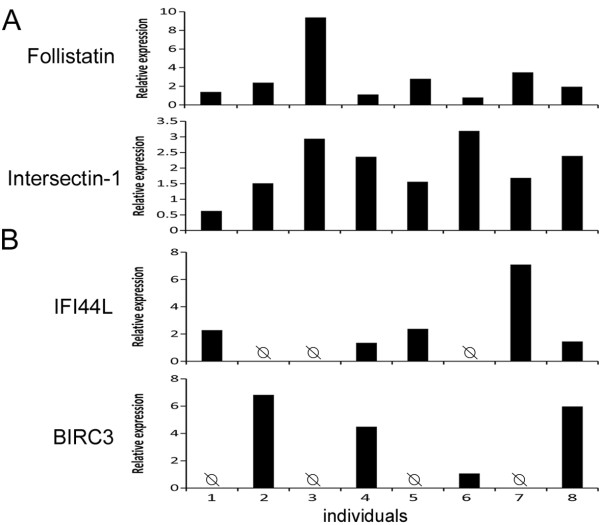
**Basal gene expression polymorphism.** Relative mRNA abundances of four hemocyte genes in eight individual oysters are shown as examples of (**A**) individual differences in basal gene expressions (*follistatin* and *intersectin-1*); (**B**) presence or absence (Ø) of basal expression for an interferon-induced 44-like protein (IFI44L) and a baculoviral inhibitor of apoptosis repeat-containing protein 3 homologue (BIRC3). Note that each individual displays a unique profile of gene expression for these four transcripts. Relative expression levels were calculated according to the 2^-ΔΔCq^ method normalized with the ribosomal L40 protein (*Cg-rpl40*).

A total of 280 hemocyte-expressed genes were chosen from the 1,049 sequences differentially expressed (>5-fold change) between SVir-8d and NSVir-8d DGE libraries. Both GenBank annotated (62 %) and non-annotated (38 %) sequences were considered. The technical efficiency of three independent 96.96 dynamic arrays was assessed by measuring the expression of the three reference genes in each qPCR chip (ANOVA test). Then, the expression profile of the selected genes was assessed to compare their mRNA abundance according to the oyster survival phenotypes (S versus NS) at the individual level. Surprisingly, the analysis of the large qPCR data set revealed an extraordinary interindividual variability in gene expressions for most of the transcripts measured. As illustrated in Figure 2A, striking inter-individual differences in basal gene expression were seen for some genes with up to 20-fold change, for example, for the *follistatin*. To our knowledge, such a high inter individual variability in basal gene expression has never been previously reported in oysters.

Additionally, the high-throughput qPCR analyses revealed numerous genes whose basal levels of expression could not be detected in some individuals (Figure 2B). Interestingly, those GenBank annotated sequences appeared to correspond to genes involved in immune responses, including an interferon-induced protein 44-like, a tumor necrosis factor ligand superfamily member 10 (TNFSF10), a member of oyster lysozymes (*Cg*_lysoz2), a baculoviral inhibitor of apoptosis repeat-containing protein 3 homologue (BIRC3) and members of the big defensin antimicrobial peptide family (*Cg*-BigDef) [24]. However, no significant relationships could be found between the expression detection of these genes and the S or NS phenotypes (ANOVA test).

### A hemocyte 14-gene expression signature as a predictor of oyster survival capacity

**Figure 3:**
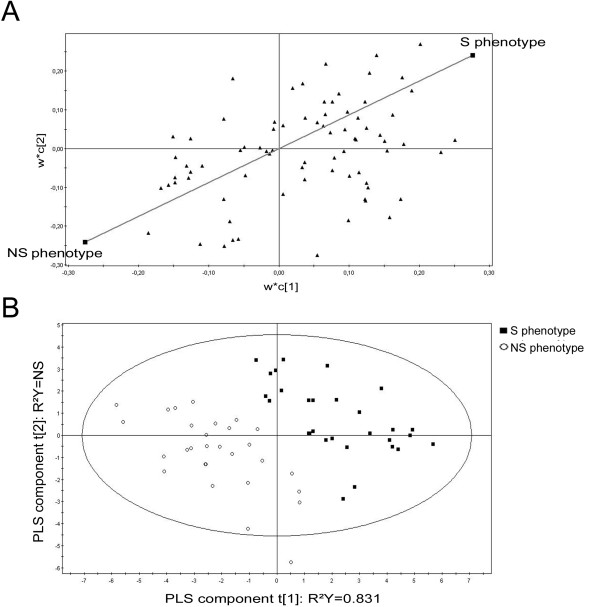
**Partial least-squares (PLS) discrimination between hemocyte-expressed genes and oyster survival phenotypes (Survival and Non-Survival).** (**A**) The graphic shows the X-loadings (w*) of the X variables (qPCR data) and the Y-loadings (c) of the Y variables (S and NS phenotypes), and thereby shows the relationship between X and Y variables. The variables X (black triangles) and Y (black squares) combine in the projections, and the variables X relate to the variables Y. (**B**) Relationship between hemocyte-expressed genes and oysters from S or NS phenotypes using PLS regression. The cross-validation led to two components represented as t[1] and t[2].

We have investigated the correlation between the profiles of hemocyte basal gene expression and the oyster survival capacity by the analysis of the mRNA abundance of genes presenting exploitable quantitative values (5 ≤ Cq ≤ 25). Due to well known oyster DNA sequence polymorphism, rigorous melting curve analysis was required to ensure qPCR specificity and avoid misinterpretations of gene expression data [25]. Thus, we only considered the qPCR data of 233 genes, from the 280 initial ones, expressed in the 45 S and 45 NS individual oysters. In spite of the high gene expression polymorphism, statistical partial least-squares (PLS) regression [26,27] analysis revealed that the basal expression levels of a core set of genes were associated to S or NS phenotypes (Figure 3A). To refine our analysis, we further considered only those genes that did not display high individual variability of basal expression among the oysters from a same phenotype. Thus, the combination of the mRNA abundance of 50 genes was identified that comprises a set of 19 genes highly transcribed in the S phenotype and a set of 31 genes highly transcribed in the NS phenotype (Additional File 1).

The expression profile of these 50 hemocyte-expressed genes (from two technical replicates) was further used to foresee oyster survival using the expression data from oysters from both survival phenotypes. The X-loadings (w*) corresponding to the data generated by qPCR and the Y-loadings (c) corresponding to the oyster S and NS phenotypes are presented in Figure 3B. The applied PLS model segregated most of the 90 individuals according to their survival capacity (R²Y = 0.831) (Figure 3B). To identify the hemocyte transcripts statistically related to each phenotype, we additionally applied appropriated fold change cut-offs (x > 1.7-fold change for overexpression and x < 0.7-fold change for underexpression). Mann–Whitney test (α = 0.09) was performed individually on the 50 genes associated to the survival phenotypes. Thus, ΔCq which are the most reproducible values were processed to assess a significant difference between S and NS survival phenotypes. Among the selected candidates, the expression profiles of 14 genes appeared to be significantly associated to an inherent capacity to survive infections (Table 1). Thus, we can presume that one individual with high probabilities to survive *Vibrio* infections transcribes at high levels the seven genes associated to the S phenotype, and concurrently at low levels the seven genes associated to the NS phenotype. Oysters from the NS phenotype (not able to survive infections) displayed an inverse pattern in gene expression profile for the survival signature. In the view of our findings, this 14-gene survival signature is able to predict the oyster survival capacity to infections by the pathogenic *V. splendidus* LGP32.

**Table 1:** **List of the hemocyte-expressed genes comprising the*Crassostrea gigas*14-gene survival signature**

***C. gigas*Sigenae contig**	**BlastX best hit**		**Expression statistics**	
	**Name [*species*]**	**E-value (% identity)**	**Level.err**	***P*-value**
**S phenotype**				
wy0aaa40yd07fm1.1.a.cg.2	Phosphoserine aminotransferase 1 [*Haliotis discus discus*]	2e-78 (66 %)	0.28622	0.03846
BQ427036.1.a.cg.2	Unknown gene product	-	0.38399	0.00302
wy0aba24ye12fm1.1.a.cg.2	Unknown gene product	-	0.55408	0.03746
wy0aba17yo09fm1.1.a.cg.2	Cystatin A2 [*Dictyostelium discoideum*]	4e-13 (52 %)	0.35958	0.02324
wy0aba10yp04fm1.1.a.cg.2	Multiple EGF-like domains 10 [*Mus musculus*]	2e-07 (33 %)	0.39222	0.08030
wy0aba11yk24fm1.1.a.cg.2	Unknown gene product	-	0.44713	0.01984
wy0aba11yc21fm1.1.a.cg.2	Unknown gene product	-	1.87451	0.08814
**NS phenotype**				
cdn37p0015o06_f.1.a.cg.2	Zinc finger HIT domain-containing protein 3 [*Danio rerio*]	2e-22 (42 %)	0.05424	0.07887
wy0aba18yb03fm1.1.a.cg.2	Unknown gene product	-	0.07867	0.02879
wy0aba9yd11fm1.1.a.cg.2	Poly(U)-specific endoribonuclease-D [*Xenopus laevis*]	9e-12 (47 %)	0.05678	0.00460
wy0aaa27yp20fm1.1.a.cg.2	Unknown gene product	-	0.05616	0.02180
cdn20p0004b10.f.1.a.cg.2	Unknown gene product	-	0.14109	0.06483
cdn20p0005d09.f.1.a.cg.2	Unknown gene product	-	0.06659	0.04859
oygd10b10d19r1_m13rev.1.a.cg.2	Cytochrome *c* [*Pectinaria gouldii*]	6e-49 (85 %)	0.08880	0.00854

### mRNA abundance of the 14-gene markers is revealing an inherent genetic trait rather than a particular physiological status

**Figure 4:**
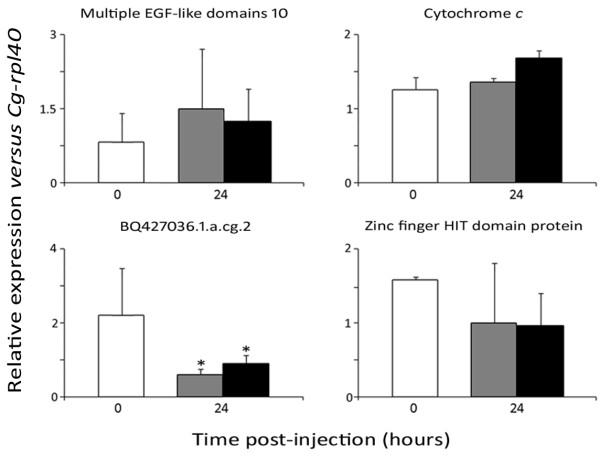
**Relative expression profile of transcripts from the 14-gene survival signature following oyster challenge.** The two types of expression profiles obtained are illustrated taking 4 genes among the signature, as examples. Hemocyte gene expression was analyzed by qPCR in unchallenged oysters (white bars) and in oysters 24 h after *Vibrio splendidus* LGP32 (black bars) or sterile sea water (grey bars) injection. Results are presented as mean values from 10 individuals per conditions. Relative expression levels were calculated according to the 2^-ΔΔCq^ method normalized with the ribosomal L40 protein (*Cg-rpl40*). Asterisks (*) indicate significant differences of gene expression between conditions (*P* < 0.05).

We confirmed that the survival signature revealed an inherent genetic trait in oysters rather than a pre-stimulation or a physiological response. Indeed, high mRNA abundance of the genes of the signature could have resulted from an increase in gene transcription for some individuals before the experiment. For that, oysters (3 pools of 10 animals/conditions) were injected with a sublethal dose of *V. splendidus* LGP32 (5 x 10^7^ CFU/animal) or sterile sea water (SSW). Non injected oysters were used as a control. As previously reported, a great variability in transcript rates was observed for both unchallenged and immune challenged (*Vibrio* or SSW) oysters. Two main types of expression profiles were obtained among the signature genes (Figure 4). Nine genes displayed no changes in transcript abundance at 24 h following either bacterial or SSW injection, compared to unchallenged animals as illustrated for *multiple EGF-like domains 10* and *cytochrome c* (Figure 4). Besides, five genes, including the unknown *BQ427036.1.a.cg2* and *zinc finger HIT domain-containing protein 3* shown in Figure 4 as examples, displayed a decrease of transcript abundance in circulating hemocytes following both the bacterial and SSW injection, thus independently of an acute response to the *Vibrio*. No up-regulation was seen for any of the 14 hemocyte-expressed genes following the bacterial challenge or SSW injection. Thus, mRNA abundance of the 14 genes of the survival signature reveals, in oysters, basal gene expression independent from their physiological response.

### Polymorphism in hemocyte basal gene expression can be associated to variations in gene copy numbers in oyster genome

**Figure 5:**
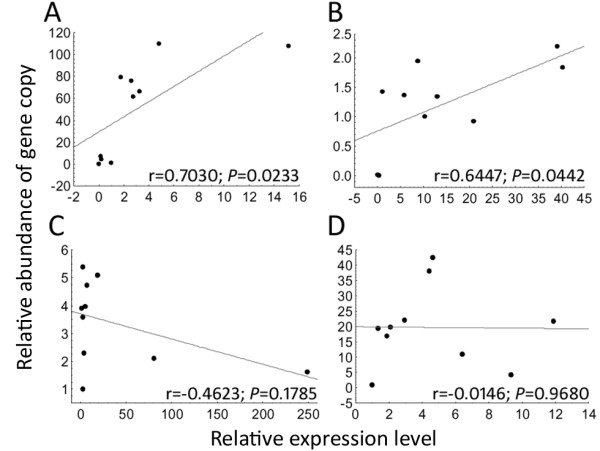
**Examples of correlations between relative abundance of gene copy and basal gene expression levels for four gene markers.** Relative expression and gene copy abundance were estimated by qPCR at individual level (n = 10) for (**A**) wy0aba11yc21fm1.1.a.cg.2, (**B**) cdn20p0004b10.f.1.a.cg.2, (**C**) Multiple EGF-like domains 10, and (**D**) wy0aba18yb03fm1.1.a.cg.2. Relative expression levels were obtained according to the 2^−ΔΔCq^ method using the *Cg-rpl40* as constitutively expressed gene. Relative abundances of gene copy were obtained with the same method but using the single copy *Cg-bpi* gene as reference. Grey line represents linear fit, significant correlations are indicate by *P* < 0.05 and r indicates the correlation coefficient.

In an attempt to understand the genetic basis involved in the transcriptional regulation of the genes associated to oyster survival, we investigated whether variability in gene expression would reflect gene copy number variations (CNV) in oyster genome. A relative quantification approach was developed to verify a potential correlation between gene copy abundance and mRNA expression of the oyster survival signature. For that, both genomic DNA and total RNA were extracted from ten oysters showing striking differences in gene expression of seven genes from the 14-gene survival signature for which pairs of primers present same efficiency and specificity on gDNA and cDNA. Variations in gene copy number were shown for all analyzed genes. Higher variations in gene copies (from 4.5 to 120-fold changes) than in expression levels (from 2.2 to 16-fold changes) were seen for four genes (as example Figures 5A and 5D). Conversely, for the other three genes, the variations in expression were higher (from 4.5 to 250-fold changes) than in gene copy number (from 2 to 6-fold changes) (as example Figures 5B and 5 C). Finally, among those seven genes, only two presented positive correlation (*p* < 0.05) between the relative abundance of gene copy and relative gene expression (Figures 5A and 5B).

## Discussion

Herein, we successfully identified a 14-gene survival signature, a cluster of hemocyte-expressed genes whose basal expression profile was predictive of the oyster capacity to survive *Vibrio* infections. This study provides the first evidence that oyster survival to infectious diseases can be driven by genetic determinants. We revealed an extraordinary interindividual polymorphism in basal gene expression that is associated to an inherent capacity of some oysters to survive *Vibrio* infections.

In previous work, we applied DGE to highlight immune functions involved in the successful response of *C. gigas* to circumvent virulent *Vibrio* strain infections compared to a non virulent one [21]. Here, the aim was to evidence gene markers of an inherent survival capacity rather than to characterize biological functions or molecular mechanisms implicated in that capacity. We just observed an enrichment of genes related to immune response and genetic information processing in the SVir-8d DGE library. The comparative analysis of the hemocyte transcriptomes of oysters able (SVir-8d) or not able (NSVir-8d) to survive did not highlight striking functional categories as potentially involved in oyster health promotion and conferring advantageous capacity to survive infections. This is not surprising considering the low number of sequences found with predicted functions (only 385 instead of 664 with unknown functions). With further progress in the genetic resources in *C. gigas*, one can return to exploit the DGE data and expect to characterize unambiguously biological functions associated with oyster fitness and optimal survival capacity.

The comprehensive and detailed quantitative analysis of the DGE data at the individual level has shown here for the first time in oysters the remarkable interindividual polymorphism in hemocyte gene expression. It is illustrated by great variations in basal mRNA levels up to 20-fold change between individuals. In human, variations in the basal mRNA abundance of some key genes represent an important genetic basis underlying several physiological traits that includes resistance (or susceptibility) to many known infectious diseases [14]. Moreover, in many oysters from both survival phenotypes, basal mRNA levels of some immune-related genes were not detected. It cannot be ruled out that due to a high level of DNA polymorphism, only transcripts encoded by particular alleles could have been amplified [25]. Indeed, it is widely recognized that oysters are one of the highest polymorphic species in the animal kingdom (one SNP every 40 bp in genomic non-coding regions) [17,28]. Moreover, most of transcripts whose expression was shown to be absent among individuals corresponded to immune-related proteins, which have been shown in *C. gigas* to display a high level of sequence diversity [29]. Another hypothesis is that some of these genes are not expressed in some individuals because of the absence of gene in the oyster genome. This has been shown for the human neutrophil antimicrobial peptide-3 (HNP-3) [30,31]. Since HNP-3 was proved to play an important role in infectious and inflammatory diseases [32], it has been suggested that the absence of *DEFA3* gene can result in changes in the human susceptibility to pathogenic microorganisms [30]. Here, probably due to limited sampling number, we did not evidence a statistical correlation between the lack of hemocyte gene expression and the oyster capacity to survive infections, but this result prompted us to further analyze this phenomenon of absence of immune-related transcripts in oyster populations.

The existence of a genetic predisposition of oyster to survive *Vibrio* infections was revealed by DGE and high-throughput qPCR analyses. Performed on independent sets of 180 oysters, from different geographical origin and history, these different transcriptomic analyses concurred in the statistical identification of a gene survival signature. Indeed, in spite of the high polymorphism of gene expression, the combination of the basal expression levels of 14 hemocyte-expressed genes was shown to be correlated to oyster capacity to survive infections by the pathogenic *V. splendidus* LGP32. This 14-gene survival signature includes 8 unknown genes and the potential role of the other annotated genes in the oyster health status and survival cannot be interpreted. However, interestingly, one member of the survival signature, a putative cystatin A, has already been identified as related to the ability of oyster to circumvent virulent *Vibrio* infection in previous genomic study [21]. Since *V. splendidus* LGP32 and *V. aestuarianus* are known to secrete extracellular proteases, we can assume that, highly expressed at basal level, cystatin, as protease inhibitor, may interact with the *Vibrio* toxins contributing to oyster survival [21]. It is noteworthy that cystatins are a superfamily of multifunctional proteins [33] that requires extensive characterization in oyster before speculating any function or role in survival. Recently, a mortality gene expression signature was identified in *C. gigas* hemocytes of oysters that died in the field during summer mortality events in USA [34]. This so-called “mortality signature” comprised a core set of genes involved in cell death, protein synthesis and cellular assembly and organization. Their expression increased in individuals previously to the occurrence of massive mortalities in the field [34]. Besides, Fleury et al. [35,36] also showed in oyster tissues, such as gonads, gills and muscle, transcripts of immune-related genes differentially expressed between two oyster lines selected for their resistance and susceptibility to the 2001 summer mortality events in France. However, these genes were identified according to an increase of their transcript abundance during various periods preceding a mortality event, revealing a physiological response of the oysters to field conditions. Instead, our study has focused on the identification of genes whose basal expression in oyster may reflect an intrinsic character of survival capacity, i.e. a genetically-based trait underlying natural variation in gene expression.

In this attempt, it was important to verify that, in our experiments, the high mRNA levels of the 14-genes identified as related to survival capacity were not due to a particular physiological status or a priming of the oysters prior to the hemolymph sampling. Indeed, priming could confer protection against *V. splendidus* LGP32 infection, as described in various invertebrate species, where pre-stimulated animals were protected against a second and lethal challenge, and in a specific manner [37,38]. Here, following microbial challenge or injury (sea water injection), no increase in transcript abundance was observed from the genes of the survival signature. Instead, a decrease of transcripts was observed for five of them. Such a decrease is either related to a migration of the hemocyte subpopulation expressing those genes towards the site of injection [39], or to a down-regulation of expression following wound injection. Besides, nine genes were evidenced as constitutively expressed in circulating hemocytes and not regulated by a microbial challenge. Ruling out a priming possibility or an effect of physiological status, these results are in favor of genetic bases driving hemocyte high basal gene expression in some oysters.

In our study, copy number variations (CNVs) in the 14 marker genes were evidenced among the individuals tested, as previously reported by Schmitt et al. [29] for genes encoding actin and two antimicrobial peptides (*Cg*-Defs and *Cg*-Prps). It is now established that CNV contributes to differences in gene expression and can be associated to variable phenotypes, including susceptibility to complex genetic disorders and infectious diseases [40,41]. For instance, in human, whereas an elevated number of copies of the β-defensin gene *DEFB103* in the East Asia population is associated to increased resistance to influenza [42], high α-defensin (*DEFA1A3*) gene copies were shown to be significantly correlated with susceptibility to severe sepsis [43]. Here, we have not found a correlation between CNV and gene expression profile for all of the genes of the survival signature. As elegantly shown by Mileyko et al. [44], the basal expression of some genes is not necessarily proportional to the gene copy number in the genome due to transcriptional feedbacks in complex regulatory networks. Because oysters are highly polymorphic [28], changes in gene expression can also be associated to a high degree of DNA polymorphism in the gene promoter sequence [45]. Our results indicated that the mRNA levels of the different genes from the 14-gene survival signature are likely driven by distinct transcriptional processes.

## Conclusions

In conclusion, our findings evidenced that oysters possess a complex genomic organization that is highly polymorphic, not only in terms of DNA sequence [28], but also in terms of number of gene copies and transcriptional regulation of genes. A deep analysis of the whole hemocyte transcriptome of oysters showing differences in the resistance to *Vibrio* infection led to the discovery of a 14-gene predictive survival signature. Currently, in human medicine, such gene expression signatures have considerably improved the diagnosis and understanding of complex genetic disorders and infectious diseases [12,15,16]. In the context of *C. gigas* aquaculture, the mass mortality syndrome represents a serious constraint to oyster production world-wide. Thus, understanding the genetic basis involved in oyster survival constitutes an important piece in the oyster-pathogen-environment interaction puzzle. Although oyster immunogenetics is still an emerging domain that requires great improvement in genomic resources, we showed that gene predictive-survival signature could provide a useful tool for preventing mortality events and for assessing genetic traits of interest for disease resistance selection programs.

## Methods

### Oysters and experimental *Vibrio* infections

For DGE library construction, 2 year-old adult *Crassostrea gigas* oysters were purchased from an Atlantic oyster farm (La Tremblade, France). Following an acclimatation period of one week in aquaria containing filtered sea water at 20 °C, 180 animals were individually tagged and hemolymph (~500 μL per animal) was withdrawn from the adductor muscle without causing the death of the animals. Hemocyte samples were individually collected by centrifugation (1,500 x *g* for 15 min at 4 °C), homogenized in 1 mL of TRIzol reagent (Invitrogen) and frozen at −20 °C until RNA extraction. After hemolymph collection, animals were placed in glass tanks (20 animals per tank) and allowed to recover for 8 days prior to experimental infections. Then, 90 oysters were intramuscularly injected with 8 x 10^7^ CFU/animal of *V. splendidus* LGP32 and a second group of 90 oysters with 2 x 10^7^ CFU/animal of *V. aestuarianus* LPi 02/41. Mortalities were monitored daily and individually tagged animals that did not survive were noted and discarded. After the end of acute mortalities (96 h post-infection), stored hemolymph samples were categorized according to oyster survival (surviving versus non-surviving).

For high-throughput qPCR analyses and discovery of candidate genes associated to oyster survival capacity, three independent infections (n = 60 oysters per experiment) were further conducted on oysters (2 year-old) from Mediterranean oyster farm (Sodimer, Balaruc, France) with *V. splendidus* LGP32 (5 x 10^8^ CFU/animal) following the same protocol as applied for DGE libraries construction. Finally, a last set of experiments was performed to control the expression modulation of the signature genes upon infection or injury. In this experiment, oysters were injected with a sublethal dose (5 x 10^7^ CFU/animal) of live *V. splendidus* LGP32. Unchallenged oysters (i.e., oyster at time 0 h) and oysters injected with 100 μL SSW (sterile sea water) were used as controls. Hemolymph was collected 24 h post-injection and hemocytes were collected and pooled (3 pools of 10 animals per condition) for RNA extraction. The standardization of the experimental infections and the preparation of the bacterial inoculum were performed as previously described [46]. All experimentations were performed according to the Ifremer animal care guideline and policy.

### DGE library construction and oyster tag-to-gene mapping

Two DGE libraries were constructed from pooled hemocyte RNA samples of oysters able (SVir-8d DGE library) and not able (NSVir-8d DGE library) to survive *Vibrio* infections, according to protocols previously described [21,47]. The obtained DGE tag signatures were matched against 29,745 unique sequences from different tissues of *C. gigas* stored on the platform of Sigenae-INRA Toulouse (http://public-contigbrowser.sigenae.org:9090/Crassostrea_gigas/index.html). For tag to gene mapping, the virtual tags were extracted from all contigs and singletons. Only tags with 100 % sequence identity with oyster EST sequences were assigned. Functional gene annotation was performed as described previously [21].

### High-throughput qPCR using 96.96 microfluidic dynamic arrays

Complementary DNAs (cDNA) were synthesized from 250 ng of total individual hemocyte RNA samples and preamplified by using the TaqMan PreAmp kit (Applied Biosystems). Candidate genes were selected based on the differential of tag occurrences between the DGE libraries (>5-fold change). Primers used in this study are listed in the Additional File 2. The eukaryotic translation elongation factor 1-alpha (*Cg-ef1α*) [GenBank: AB122066], the ribosomal protein L40 (*Cg-rpl40*) [GenBank: FP004478], and the 40 S ribosomal protein S6 (*Cg-rps6*) [GenBank: CU686508] were used as endogenous reference genes. The relative gene expression was measured by using the high-throughput microfluidic qPCR platform BioMark^TM^ (Fluidigm) [22]. The cycling program used consisted of 10 min at 95 °C followed by 40 cycles of 95 °C for 15 s and 1 min at 60 °C. Melting curves analysis was performed after completed qPCR collecting fluorescence between 60-95 °C at 0.5 °C increments. qPCR data were analyzed using the BioMark^TM^ qPCR analysis software (Fluidigm).

### Data analysis and statistical modeling

Expression of the three reference genes was quantified in each array chip in order to assess the technical efficiency of the qPCR experiments. Variations in Cq values associated to each qPCR experiment were evaluated using ANOVA among the reference genes. The delta Cq values (ΔCq) were determined for all target genes by subtracting the Cq values from the geometric mean of the Cq values of the three reference genes. Thus, a data matrix was built up based on the ΔCq values obtained from all qPCR dynamic arrays.

Partial least-squares (PLS) models using PLS regression were used to discriminate oyster survival on the basis of ΔCq values of the target genes. PLS predictive models were assessed using the SIMCA-P software 9.0 (Umetri, Umeå, Sweden), as previously described [26,27]. For our analysis, ΔCq values from qPCR assays and individual oysters with contrasting survival capacities were considered as variables X and Y, respectively. The PLS regression between variables X and variables Y yielded the PLS components t[1], t[2], …, t[n], which are linear combinations of variables X (t[1] = w*_11_X_1_ + w*_12_X_2_ + … + w*_1n_X_n_, t[2] = w*_21_X_1_ + w*_22_X_2_ + … + w*_2n_X_n_, t[n] = w*_n1_X_1_ + w*_n2_X_2_ + … + w*_nn_X_n_). These components describe the variables X and explain the variables Y. The number of useful PLS components was determined by cross-validation (SIMCA-P 9.0, 2001). Regression relating the variables Y to the PLS components t[1], t[2], …, t[n] are then build as follows: Y1 = C_11_t[1] + C_12_t[2] + … + C_1n_t[n] + residuals, Y2 = C_21_t[1] + C_22_t[2] + … + C_2n_t[n] + residuals, Yn = C_n1_t[1] + C_n2_t[2] + … + C_nn_t[n] + residuals. The X-loadings and the Y-loadings are noted w* and c, respectively. The explanatory performance of a model is evaluated using the R² coefficient which corresponds to the part of the variance of variables Y explained by the variables X.

The ΔCq data matrix was used for calculation of relative expression (fold change) using the 2^-ΔΔCq^ method (assuming 100 % amplification efficiency) [48]. For that, the “NS phenotype” was chosen as reference group. In order to obtain a second normalized value (ΔΔCq), the geometric mean of the ΔCq values from the reference group was subtracted from the geometric mean of the ΔCq values from “S phenotype”. Then, the relative expression ratio was calculated by using the 2^-ΔΔCq^ formula. Various fold-change and raw Cq cut-off to assess the value of the sensibility of the RT-qPCR experimentation were applied. Then Mann–Whitney tests on the ΔCq were used to assert a significant difference between S and NS phenotypes.

### Relative gene copy number estimation

Gene copy number estimation and relative expression of seven genes comprising the 14-gene survival signature were carried out using LightCycler 480 qPCR system (Roche) in a 384 well plate format. Both genomic DNA and total RNA was extracted from 10 individual oysters as described previously [29]. Each qPCR experiment was performed in triplicates and run under the following conditions: 95 °C for 10 min and then 40 cycles of denaturation at 95 °C for 10 s, annealing at 57 °C for 20 s and extension at 72 °C for 25 s, following melting curve determination. Relative expression levels were obtained using the 2^−ΔΔCq^ method using the *Cg-rpl40* as constitutively expressed reference gene. Relative abundance of gene copy was obtained with the same method but using a single copy encoded gene (*C. gigas bactericidal permeability-increasing proteinCg-bpi*) as reference [GenBank: AY165040]. Correlations between relative abundance of gene copy and relative expression were obtained by using a correlation matrix from Statistica software (V7.1, Statsoft), and significant correlations were indicated by a *p* < 0.05.

## Abbreviations

CFU, colony forming units; CNV, gene copy number variation; DGE, digital gene expression; EST, expressed sequence tags; NSVir-8d, non-surviving DGE library; PLS, partial least-squares; qPCR, quantitative real-time Polymerase Chain Reaction; SNP, single nucleotide polymorphism; SVir-8d, surviving DGE library.

## Competing interests

The authors declare no conflict of interest.

## Authors’ contributions

RDR, JdL, DP and EB designed research. RDR, JdL and DP performed research. RDR, JdL, PT, RB and DP contributed new reagents/analytic tools. RDR, JdL, PT, RB, DP and EB analyzed data. RDR, DP and EB wrote the paper. All authors read and approved the final manuscript.

## Supplementary Material

Additional file 1 List of hemocyte-expressed genes associated to oyster survival phenotypes. Click here for file

Additional file 2 Nucleotide sequences of primers used. Click here for file
