# Supplementary material for: A hemocyte gene expression signature correlated with predictive capacity of oysters to survive Vibrio infections
Source: BMC Genomics. 2012 Jun 18;13:252. doi: 10.1186/1471-2164-13-252 (PMC3418554; doi:10.1186/1471-2164-13-252)
Supplement: Additional file 1 — List of hemocyte-expressed genes associated to oyster survival phenotypes. [file 1471-2164-13-252-S1.pdf]

Additional file 1. Hemocyte-expressed genes associated to oyster Survival (S) and Non Survival (NS) phenotypes.

C. gigas Sigenae contig	BlastX best hit<br>Name [*species*]	E-value<br>(% identity)
S phenotype		
cdn37p0004a18.f.1.a.cg.2	Alpha2-macroglobulin [*Chlamys farreri*]	1e-79 (45%)
wy0aba17yo09fm1.1.a.cg.2	Cystatin A2 [*Dictyostelium discoideum*]	4e-13 (52%)
wy0aba12yp08fm1.1.a.cg.2	Cystatin B-like protein [*Crassostrea gigas*]	4e-34 (74%)
wy0aba22yf07fm1.1.a.cg.2	Integrin alpha-4 [*Mus musculus*]	9e-33 (37%)
cdn20p0005n15.f.1.a.cg.2	Metallothionein IV [*Crassostrea gigas*]	0 (100%)
cdn37p0031k04_f.1.a.cg.2	Mitochondrial glutamyl-tRNA synthetase [*Glossina morsitans*]	2e-35 (40%)
wy0aba10yp04fm1.1.a.cg.2	Multiple EGF-like domains 10 [*Mus musculus*]	2e-07 (33%)
wy0aaa40yd07fm1.1.a.cg.2	Phosphoserine aminotransferase 1 [*Haliotis discus discus*]	2e-78 (66%)
wy0aba36yl08fm1.1.a.cg.2	Proline rich peptide (*Cg*-PRPC) [*Crassostrea gigas*]	0 (100%)
wy0aba23yg22fm1.1.a.cg.2	Proline rich peptide (*Cg*-PRPL) [*Crassostrea gigas*]	0 (100%)
wy0aaa11yi05fm1.1.a.cg.2	Toll-like receptor 1 (*Cg*Toll-1) [*Crassostrea gigas*]	0 (100%)
cdn37p0007o21_f.1.a.cg.2	Universal stress protein [*Schistosoma japonicum*]	7e-19 (39%)
BQ427036.1.a.cg.2	Unknown gene product	-
oygd09b07a21r1_m13rev.1.a.cg.2	Unknown gene product	-
wy0aaa22yi02fm1.1.a.cg.2	Unknown gene product	-
wy0aba11yc21fm1.1.a.cg.2	Unknown gene product	-
wy0aba11yk24fm1.1.a.cg.2	Unknown gene product	-
wy0aba24ye12fm1.1.a.cg.2	Unknown gene product	-
wy0aba42yg14fm1.1.a.cg.2	Unknown gene product	-
NS phenotype		
wy0aaa20yi09fm1.1.a.cg.2	C-type lectin 2 like protein [*Crassostrea gigas*]	0 (100%)
oygd09b07l03r1_m13rev.1.a.cg.2	Calreticulin (CRT) [*Crassostrea gigas*]	0 (100%)
wy0aaa31yh04fm1.1.a.cg.2	Catalase [*Crassostrea gigas*]	0 (100%)
oygd10b10d19r1_m13rev.1.a.cg.2	Cytochrome *c* [*Pectinaria gouldii*]	6e-49 (85%)
wy0aaa20yc12fm1.1.a.cg.2	Early growth response protein 1 (Egr1) [*Ovis aries*]	6e-39 (68%)
wy0aaa36yl22fm1.1.a.cg.2	Intersectin 1 (SH3 domain protein) [*Danio rerio*]	2e-82 (60%)
wy0aba27yj22fm1.1.a.cg.2	Kazal-type serine protease inhibitor [*Pinctada fucata*]	1e-14 (50%)
wy0aba16yo20fm1.1.a.cg.2	L-rhamnose-binding lectin [*Hydractinia symbiolongicarpus*]	7e-17 (44%)
cdn20p0004c14.f.1.a.cg.2	LPS induced TNF-alpha factor (*Cg*-LITAF) [*Crassostrea gigas*]	0 (100%)
AB179775.1.a.cg.2	Lysozyme (CGL-1) [*Crassostrea gigas*]	0 (100%)
cdn37p0002b19.f.1.a.cg.2	Mediator of RNA polymerase II transcription 15 [*Xenopus laevis*]	2e-60 (51%)
cdn37p0002d10.f.1.a.cg.2	MyD88 adaptor (*Cg*-MyD88) [*Crassostrea gigas*]	0 (100%)
wy0aba9yd11fm1.1.a.cg.2	Poly(U)-specific endoribonuclease-D [*Xenopus laevis*]	9e-12 (47%)
oyge10b14d14r1_m13rev.1.a.cg.2	Retinoblastoma-binding protein 4 (rbbp4) [*Aedes aegypti*]	0 (92%)
cdn19p0004m04.f.1.a.cg.2	Tetraspanin 33 [*Danio rerio*]	5e-11 (37%)
wy0aaa18yn15fm1.1.a.cg.2	Tissue inhibitor of metalloproteinase [*Crassostrea gigas*]	0 (100%)
cdn37p0012m10_f.1.a.cg.2	Trans-2,3-enoyl-CoA reductase [*Xenopus laevis*]	5e-28 (48%)
cdn37p0022h15_f.1.a.cg.2	Vacuolar-sorting protein SNF8 [*Ascaris suum*]	2e-66 (59%)
cdn37p0015o06_f.1.a.cg.2	Zinc finger HIT domain-containing protein 3 [*Danio rerio*]	2e-22 (42%)
cdn20p0004b10.f.1.a.cg.2	Unknown gene product	-
cdn20p0005d09.f.1.a.cg.2	Unknown gene product	-
cdn21p0004f02.f.1.a.cg.2	Unknown gene product	-
cdn37p0009d10_f.1.a.cg.2	Unknown gene product	-
oypg09b06c09r1_m13rev.1.a.cg.2	Unknown gene product	-
oyge09b12i23r1_m13rev.1.a.cg.2	Unknown gene product	-
oyge10b14n09r1_m13rev.1.a.cg.2	Unknown gene product	-
wy0aaa27yp20fm1.1.a.cg.2	Unknown gene product	-
wy0aaa13yh23fm1.1.a.cg.2	Unknown gene product	-
wy0aaa33ym17fm1.1.a.cg.2	Unknown gene product	-
wy0aba18yb03fm1.1.a.cg.2	Unknown gene product	-
wy0aba22yf10fm1.1.a.cg.2	Unknown gene product	-
