# Supplementary material for: A hemocyte gene expression signature correlated with predictive capacity of oysters to survive Vibrio infections
Source: BMC Genomics. 2012 Jun 18;13:252. doi: 10.1186/1471-2164-13-252 (PMC3418554; doi:10.1186/1471-2164-13-252)
Supplement: Additional file 2 — Nucleotide sequences of primers used. [file 1471-2164-13-252-S2.pdf]

Additional file 2

| *Crassostrea gigas* Sigenae contig | **BlastX best hit** **Name** [*species*: GenBank accession number] | **E-value (% identity)** | **Primer Fw** | **Primer Rv** |
|---|---|---|---|---|
| oygd09b08j18r1_m13rev.1.a.cg.2 | *Crassostrea gigas* elongation factor 1 alpha (*Cg-ef1α*) [*Crassostrea gigas*: BAD15289] | 0 (100%) | GAGCGTGAACGTGGTATCAC | ACAGCACAGTCAGCCTGTGA |
| wy0aba23yi23fm1.1.a.cg.2 | Ribosomal protein L40 (*Cg-rpl40*) [*Crassostrea hongkongensis*: ADB22377] | 3e-68 (99%) | AATCTTGCACCGTCATGCAG | AATCAATCTCTGCTGATCTGG |
| oypm10b04m14r1_m13rev.1.a.cg.2 | 40S ribosomal protein S6 (*Cg-rps6*) [*Pinctada maxima*: ACS72283] | 3e-92 (91%) | CAGAAGTGCCAGCTGACAGTC | AGAAGCAATCTCACACGGAC |
| wy0aba36ya14fm1.1.a.cg.2 | Unknown gene product | - | AGGCAATGGTTGATCGTGTG | CTGAATCCGTACATGGATGC |
| wy0aba12yg08fm1.1.a.cg.2 | Unknown gene product | - | AAATGGCCAACAAACTCCAG | GACTTCAGCCCTGATGGTTC |
| cdn37p0014h22_f.1.a.cg.2 | TBC1 domain family member 9 [*Harpegnathos saltator*: EFN77590] | 2e-23 (35%) | CTCAAGCTCATCTCAGTCAG | ATTGACCAATCGGAGTCTGG |
| oypg09b05e24r1_m13rev.1.a.cg.2 | Mitochondrial ATP synthase B subunit [*Clonorchis sinensis*: ABI78941] | 1e-22 (29%) | CATCTGTACAGGCAACAGGC | GGTAGTGGACATTGCTCCAG |
| wy0aba10yp04fm1.1.a.cg.2 | Multiple EGF-like-domains 10 (MEGF10) [*Mus musculus*: EDL09867] | 2e-07 (33%) | TGACGTGCTCGGCAACATGC | CATTGTGTCCCCGTGAAGCC |
| cdn37p0010a02_f.1.a.cg.2 | Unknown gene product | - | GGCTCCAATAACTGAAAGTG | CACATACCTTCTTGGTAACC |
| cdn37p0031a15_f.1.a.cg.2 | NADH dehydrogenase (ubiquinone) flavoprotein 2 [*Schistosoma japonicum*: CAX75944] | 2e-90 (65%) | GCCATGCACAAAGTAGCAGA | CTACTCCGCCCAGGATACAG |
| cdn37p0005l08.f.1.a.cg.2 | Nascent polypeptide-associated complex alpha polypeptide [*Oreochromis niloticus*: AAN86982] | 5e-56 (72%) | CCTGTAGAAGACACCAAGCC | GCTCTGTTGCTGAAGGTCAC |
| wy0aba27yj22fm1.1.a.cg.2 | Kazal-type serine protease inhibitor [*Pinctada fucata*: ADC52432] | 1e-14 (50%) | CCATATCTAACGATGGTGCC | CTATAATCTGCTGTGCACGC |
| cdn37p0033i22_f.1.a.cg.2 | Unknown gene product | - | CAAGGGCTGTTCACAAACTG | TCTGACTTGGCTGTTGTTCC |
| wy0aba41yp10fm1.1.a.cg.2 | Unknown gene product | - | CAATGCCAATACAACGACC | TGTCTTCCCAAACACATGG |
| wy0aaa12yb09fm1.1.a.cg.2 | Sodium/glucose cotransporter 4 [*Danio rerio*: NP_001107077] | 2e-64 (62%) | GCGCTGATGAGTTCTCTGAC | GATCAGGATGAACACTCTGC |
| cdn37p0004i11.f.1.a.cg.2 | Unknown gene product | - | GCAGGATGTATGTGAGCTTG | CCCTTGACCAGTAGATGCAC |
| cdn37p0016n16_f.1.a.cg.2 | Unknown gene product | - | GGAATGGAACAAACGCCTTG | ACAGGAAGGTCGTCTTCATC |
| wy0aba33yb16fm1.1.a.cg.2 | Unknown gene product | - | GTCGCCCACTATAATTACCG | TCCTAGATAGCCGCAGCTC |
| cdn19p0003g15.f.1.a.cg.2 | Serine/threonine-protein phosphatase 4 catalytic subunit (PPP4C) [*Harpegnathos saltator*: EFN85419] | 2e-170 (94%) | GATTCCTGTAGTTGACAGATG | GCGTCAACTGATGGAAGAAG |
| oyge10b14c18R1_m13rev.1.a.cg.2 | Nucleoporin Nup153 homolog [*Xenopus laevis*: AAC41273] | 2e-08 (53%) | AGTCTGCGCGCCTAATATG | CTCCAGATTTAGCTTCCTAAC |
| wy0aba31yb03fm1.1.a.cg.2 | Unknown gene product | - | AGAATGTCTGGACGCGGAC | CCTTCAGGTTTATTGCCACG |
| wy0aba12yh03fm1.1.a.cg.2 | Unknown gene product | - | CCCTAGCCAAGATGGCATG | GTCTTCCGGCTATTATACGC |
| cdn21p0004d04.f.1.a.cg.2 | SH3 domain-binding protein 2 [*Gallus gallus*: NP_001026336] | 2e-07 (25%) | GCATTCCTGTCAGTCTACTG | ACTGTCTATAGCGTCATGCC |
| wy0aaa21yo12fm1.1.a.cg.2 | Unknown gene product | - | CACCTGTCTCACCTACTTAC | CACATCAATTCACTGGGTTAG |
| wy0aba38ym18fm1.1.a.cg.2 | Unknown gene product | - | TGTGGACATGCTGTTAGTCG | CTACGTGAGATATAGACCCG |

Additional file 2

| | | | | |
|---|---|---|---|---|
| cdn21p0001k05.f.1.a.cg.2 | Unknown gene product | - | TAGGCGTTGTCCCAAAGCC | CACAATGCACAGTTGCGTG |
| wy0aba14yh02fm1.1.a.cg.2 | Unknown gene product | - | TCGGACATTTCAGTAGCGTG | CAGAACCACTGCTACCGTC |
| wy0aaa38yp16fm1.1.a.cg.2 | Unknown gene product | - | AGGTAGACGGATGATTGCTG | CAAGTCACCACTATCTGGAG |
| oyge09b12i23r1_m13rev.1.a.cg.2 | Unknown gene product | - | CGACGTGTATGTTCGACACA | CTGCGATCTACATCTAGCTC |
| cdn37p0009d10_f.1.a.cg.2 | Unknown gene product | - | CAGTGTAGGAAGAATCCTCG | CCCGTGTGTTACCTTCAGC |
| cdn37p0016k21_f.1.a.cg.2 | Unknown gene product | - | GAGAGTGCAGTGAACAGCTA | CACATTGCAGCTTAATCCTC |
| cdn21p0004e05.f.1.a.cg.2 | Ribonuclease kappa-A [*Danio rerio*: NP_001038870] | 8e-20 (50%) | TTCAGACTAGGAGTCACAGG | CAGTTGTAGGCTGTTTGGTC |
| wy0aba35yn15fm1.1.a.cg.2 | Serine dehydratase [*Aplysia californica*: AAU05774] | 2e-18 (63%) | ATGCAGATGACCATCGACAG | AAGGTCATTGAGGATGCCAC |
| oygd09b08l04r1_m13rev.1.a.cg.2 | Unknown gene product | - | CCCATCCAGTTACAGAGAGC | GATTCCGCGATGACATCAGC |
| cdn19p0003o14.f.1.a.cg.2 | 26S proteasome non-ATPase regulatory subunit 6 [*Harpegnathos saltator*: EFN86625] | 4e-163 (71%) | GAGGCAAACTGTATGGCTCC | CTAACTTCGGTCTCTCCTAG |
| wy0aaa30yi19fm1.1.a.cg.2 | Unknown gene product | - | GCTGCTGAGTAAAGTGATGG | ATCAGGTATCCAACAATGCC |
| cdn37p0008i12_f.1.a.cg.2 | Unknown gene product | - | AGGCAGAACATGCAGCAGAG | TCACGACGTCTGTTGTAGTC |
| cdn37p0008l11_f.1.a.cg.2 | Unknown gene product | - | GTACTCGTCTGTTGGTAGTC | TAATCCCACTCGCAGTTCAG |
| wy0aaa27yp20fm1.1.a.cg.2 | Unknown gene product | - | AGTGTTGTAGGTCCACATGC | TGATACCAGGCAGATCGATG |
| wy0aba10yd22fm1.1.a.cg.2 | Allograft inflammatory factor 1 (AIF-1) [*Suberites domuncula*: CAC38780] | 7e-53 (74%) | GCTAAACCCACAGGTCTGC | TCATATCGCAACACACGTCG |
| wy0aba32yp17fm1.1.a.cg.2 | Microsomal glutathione S-transferase (MGST1) [*Venerupis philippinarum*: ACU83223] | 4e-30 (50%) | TGGCGATGCTTAGTTTGGAT | ATCCTCTCCACACGCTCATC |
| wy0aba24ye12fm1.1.a.cg.2 | Unknown gene product | - | TCGTAGAACCAAAGACGGAG | TGGTCTTGTAAACGAGCACG |
| oygd10b10n03r1_m13rev.1.a.cg.2 | Unknown gene product | - | CAAAGACGAGAGCGCAGCA | AACTCTCCATTCAGTGGCAG |
| cdn37p0006f10.f.1.a.cg.2 | Dihydropteridine reductase [*Harpegnathos saltator*: EFN84426] | 1e-74 (63%) | TTGTTGCTCTGTTATGCTG | GAATGTCCAACCAAGTGTG |
| wy0aaa1ye06fm1.1.a.cg.2 | Unknown gene product | - | AACCAAGTACATGTATCTGC | TCCTGGATACGAACAGCATC |
| wy0aba10yj22fm1.1.a.cg.2 | Unknown gene product | - | GAATGAACCCAAGGAGTTCC | AATTGTATCTGCTGGGCTGC |
| cdn19p0003o06.f.1.a.cg.2 | Small nuclear ribonucleoprotein Sm D3 [*Harpegnathos saltator*: EFN75405] | 4e-45 (88%) | CATGTATCAGAATCGTCGTC | ATGAACACGATGTGCGCAG |
| wy0aaa13yh23fm1.1.a.cg.2 | Unknown gene product | - | GGTAAAGTCCATTCGCTCGA | GGATTTCAGATACAGCTGAG |
| cdn37p0002o19.f.1.a.cg.2 | 60S ribosomal protein L38 [*Salmo salar*: ACN10033] | 5e-18 (77%) | GCTTCAGAATGCCAAAGCAG | ACAGGTATCGGCTACATCTG |
| wy0aba10yo14fm1.1.a.cg.2 | Unknown gene product | - | CGATGTTTGTCTGTCCATCG | GTACACTCGCATCATGTGAC |
| wy0aba11yc21fm1.1.a.cg.2 | Unknown gene product | - | CAATTACGTACGAGAGTCGC | TAGACACGATTGCACAGACG |
| cdn37p0019k05_f.1.a.cg.2 | Alpha-N-acetylgalactosaminidase [*Camponotus floridanus*: EFN70460] | 2e-48 (49%) | ACCTAACAAACTGGTAGCTG | TGTTACAGCTCTTGGCGATG |
| oyih10b03m03r1_m13r.1.a.cg.2 | Unknown gene product | - | CCACTGGACTACATTTGTAC | CAACCCCTTCTCATCATAGG |
| wy0aaa38yk21fm1.1.a.cg.2 | 3-oxoacyl-[acyl-carrier-protein] reductase [*Culex quinquefasciatus*: | 4e-84 (67%) | TGGCAGTTCCTCATCTGATC | TGCAAGCTCAAGCGCTGTG |

| | | | | |
|---|---|---|---|---|
| | XP_001863196] | | | |
| wy0aba18yd23fm1.1.a.cg.2 | Unknown gene product | - | CTGGTTTCCTAGCCAACATG | GCAGCATTACACATTGCAGC |
| CX068974.1.a.cg.2 | Unknown gene product | - | ATTGATCATCGCATGGCAGA | CATGCTCTAATGATCAAGCC |
| wy0aba28ym08fm1.1.a.cg.2 | Inhibitor of apoptosis protein-1 (IAP-1) [*Homo sapiens*: AAC83232] | 2e-09 (40%) | GGATATTCCGAATGAGATCGA | TTGACAGCAAGTGCACAGTC |
| BQ427351.1.a.cg.2 | Unknown gene product | - | GTCCCAAATCAGGACATAGC | GATGACCACCAATGTGTTCG |
| wy0aaa12yg08fm1.1.a.cg.2 | Unknown gene product | - | TACCTTGGTCAGCAGTATGC | CGCATCTCTATTTACGTTGAC |
| cdn20p0005n15.f.1.a.cg.2 | *Crassostrea gigas* metallothionein IV (*Cg*MT4) [*Crassostrea gigas*: CAK22381] | 0 (100%) | CAGCTCACACAGTCCCTTC | CATGTACAGTTACACGATGC |
| wy0aba22ym19fm1.1.a.cg.2 | PREDICTED: *Crassostrea gigas* putative inducted cysteine-rich protein | 0 (100%) | TTTGTGCCACTGAAGCCTTG | GTCCCTATACAGCAGAAGAG |
| cdn37p0006b08.f.1.a.cg.2 | NADH dehydrogenase [ubiquinone] iron-sulfur protein 8, mitochondrial precursor [*Pongo abelii*: NP_001125353] | 2e-79 (74%) | GATGCTATTGTTGAGGGTCC | CACTTGTCTCCATTGCTCAG |
| cdn19p0004m04.f.1.a.cg.2 | Tetraspanin 33 [*Danio rerio*: CAI20758] | 5e-11 (37%) | TGACGTAACCTTAGCTACCG | ATAGCCTACATCCGTGTCTG |
| cdn20p0005d09.f.1.a.cg.2 | Unknown gene product | - | CTGGTCACAGTGGAAGCAG | CACACGATGTCATGTTCTCG |
| oyge10b14n09r1_m13rev.1.a.cg.2 | Unknown gene product | - | TTGACCGAACCCCAAACCAC | GGCCTACGTCATAATGCATG |
| wy0aba42yb21fm1.1.a.cg.2 | Putative C1q domain containing protein MgC1q58 [*Mytilus galloprovincialis*: CBX41707] | 1e-21 (43%) | GGATGGAAATTCAGTGGGTC | ACTGATAACAGCTGCAGTCC |
| ES789131.1.a.cg.2 | Unknown gene product | - | TGCAGCTGAATTACGGCATG | CATGCCATATAGCTGCAGTG |
| cdn37p0016p04_f.1.a.cg.2 | Unknown gene product | - | GGCCTCCGAACTGAAGATTG | TAGCCATTGACACTCGGCAG |
| cdn21p0001k03.f.1.a.cg.2 | Unknown gene product | - | CGTTTCGGAGCGATTCTGC | AGACTTTGAACCATGCGTGC |
| wy0aaa11ym18fm1.1.a.cg.2 | Unknown gene product | - | TACCTCCCTGCTGGGTTATC | ATGTGTCTCACTGACGGCAG |
| cdn21p0004c09.f.1.a.cg.2 | Unknown gene product | - | GATTTGTCCATTTGCTTGTCC | ATCTGTATAACCTACCACAGC |
| cdn37p0025k15_f.1.a.cg.2 | Mitochondrial intermembrane space import and assembly protein 40 [*Salmo salar*: ACI69535] | 3e-21 (33%) | TCACCAAGGATGAGATGTCC | AGCTCACCATTAGGTTGCAC |
| AM237776.1.a.cg.2 | Unknown gene product | - | AGGTACTCTTGGCCACTGAG | ACATGACCAGGACCATCCAG |
| BQ427259.1.a.cg.2 | Serine/threonine-protein kinase PINK1, putative [*Pediculus humanus corporis*: XP_002432225] | 6e-27 (35%) | CCTCTTCCTAGTCGAGTTCC | CTTCACAAGTATCTATCCAC |
| wy0aba18yb03fm1.1.a.cg.2 | Unknown gene product | - | CGCGCAATTAGAAGAGTCAG | GAACATCGTCAGCTGATCGC |
| wy0aba31yp11fm1.1.a.cg.2 | SAM-dependent methyltransferase [*Saccharopolyspora erythraea*: CAM03984] | 3e-07 (32%) | GGGTTACGAAGATCGGGAG | GACTTGATTTCGTCGACAGG |
| cdn37p0022k11_f.1.a.cg.2 | Unknown gene product | - | CCATTCTTCAGCTATGTAGGA | CGCGAAGTAAGTAGCACTAC |
| BQ426839.1.a.cg.2 | Unknown gene product | - | TTTACATGACGGTCAGCTCC | ACAATCCTACACGTGTGTCC |
| oypg10b08h12r1_m13rev.1.a.cg.2 | Unknown gene product | - | CAGTGTATCTCAAAGAGTCC | AGTTCTGGCCATCAAACAGC |
| oygd10b10d19r1_m13rev.1.a.cg.2 | Cytochrome c [*Pectinaria gouldii*: AAS48105] | 6e-49 (85%) | GAGGACATCTGATTGCTTGC | TGTTACGGCAAGTCAACTGC |

Additional file 2

| | | | | |
|---|---|---|---|---|
| wy0aaa30yp06fm1.1.a.cg.2 | Unknown gene product | - | AGAATGCCTACAGTCTCTCC | TAATCCTGGTGCCTATATCC |
| wy0aba23yh15fm1.1.a.cg.2 | Unknown gene product | - | CTGCATTGGATACACCTGTC | TGAAGACGATACACAGGGAC |
| wy0aba38yk10fm1.1.a.cg.2 | Unknown gene product | - | TTGCGCAATGTGTGAACAGC | TCAAGGTCTGTGCTTGTGTC |
| cdn37p0004g21_f.1.a.cg.2 | Unknown gene product | - | TGCAACATCGTTTCCATCCG | TACGAGGGAACAAGACTGAC |
| wy0aaa19yp16fm1.1.a.cg.2 | Unknown gene product | - | CATACAACCCATACAGTCGC | TTTCTGGGTCTGCTTCAGAC |
| wy0aaa9yg20fm1.1.a.cg.2 | Unknown gene product | - | TTGCCGGAACTGAACATTGC | AGCCTTCTCGGATGTTAGTG |
| cdn20p0004i03.f.1.a.cg.2 | Unknown gene product | - | TTGCCCATTACGATCACTGG | TGCCAGATTCAACACAGCAC |
| wy0aba22yj09fm1.1.a.cg.2 | Unknown gene product | - | CCAATACTCACATGTGGACC | TCTTTCGGTCCAGAGAACTG |
| wy0aba42yg14fm1.1.a.cg.2 | Unknown gene product | - | CAAGTACCTGCTTCAAGGAG | GTGTACCTAGATGATCGTGG |
| wy0aaa14yn19fm1.1.a.cg.2 | Follistatin-related protein FLRG [*Homo sapiens*: AAC64321] | 7e-10 (32%) | TGGATTCACCCCAAAGTCTC | AAGTGTGCATGACGCTACTC |
| cdn37p0002b19.f.1.a.cg.2 | Mediator of RNA polymerase II transcription subunit 15 [*Xenopus laevis*: NP_001082292] | 2e-60 (51%) | GGAATGCAACAACAACGACC | GAACTTGCATTAGGACCAGC |
| oyge09b11b11r1_m13rev.1.a.cg.2 | Unknown gene product | - | TCTGCTCCTGATTTCGTCAG | ATATGGAGGATCTGGGCATG |
| cdn19p0002m18.f.1.a.cg.2 | Unknown gene product | - | CAGAACCTCAAGGTGATAGC | AACTCAGACATGGTGTCAGG |
| oygd10b09n13r1_m13rev.1.a.cg.2 | Unknown gene product | - | AGGAGGAAATCTAGCAGTGC | GTAACAGATAGGAACGCACC |
| oypg10b08c06r1_m13rev.1.a.cg.2 | RNA binding protein yantar [*Aplysia californica*: AAQ67660] | 3e-19 (63%) | ATTGCCTCAGGATGAAGCAG | TCAATCAGTGGGCCTTGATG |
| oypg09b05b15r1_m13rev.1.a.cg.2 | Unknown gene product | - | AAGATGCCAGCCTATTCACC | GTAAGCCAATCCAAGCACTG |
| oypg09b05c06r1_m13rev.1.a.cg.2 | Unknown gene product | - | GAAGGTCCTCATAGTGTCTG | CTGTTGGCATGGTAACTGTG |
| cdn37p0013j09_f.1.a.cg.2 | Unknown gene product | - | TTGTCTGGCTATCAAGCAGG | GATCCTATCGACTTGACTGC |
| wy0aba22yf10fm1.1.a.cg.2 | Unknown gene product | - | TTTGAAGGTGTGTGGAGGAC | ACTGCATAGTGTTATTCCGTC |
| cdn21p0003h19.f.1.a.cg.2 | Unknown gene product | - | AATCCAGAAACTCCCGACTC | TGCAGTAGATATGGTTGCATG |
| cdn37p0022d14_f.1.a.cg.2 | Unknown gene product | - | TTAGGGCAATGCAGAGACCA | AACAGAGTTGGTCTCAGCTG |
| cdn37p0034n21_f.1.a.cg.2 | Unknown gene product | - | TGTAACACGGTACAAGGGAC | ATAGACAAGTTACCGAGGGC |
| cdn37p0006c11.f.1.a.cg.2 | Unknown gene product | - | CAATGAGGTCGGTTTCGCTC | AAACGCATGTGCCTGTCCAC |
| cdn37p0007o21_f.1.a.cg.2 | Universal stress protein (Usp) [*Schistosoma japonicum*: CAX70901] | 7e-19 (39%) | TTGAGGTTTCCGTGAACGAG | AACAATCACCGGAACTGACG |
| wy0aba17yo09fm1.1.a.cg.2 | Cystatin A2 [*Dictyostelium discoideum*: XP_641175 ] | 4e-13 (52%) | GCACGTTTACCTGCTGATGA | AAATGCTTATCATTTGTAAACAAGGA |
| cdn37p0011e02_f.1.a.cg.2 | Unknown gene product | - | GGCAAATCCATGGTAAGTCG | TGACTCATCATAGAGCTCCC |
| cdn21p0001i20.f.1.a.cg.2 | Unknown gene product | - | AGTGTGATTTTGTTGATGGTC | TGCTGTGGAAGACTTGAGTG |
| wy0aaa28yn24fm1.1.a.cg.2 | Unknown gene product | - | CAGGTGTCGATGACTGTAAC | TGCTCCGTAAACACAGTCTC |
| wy0aba11yk24fm1.1.a.cg.2 | Unknown gene product | - | AGGCGATGGTTACAGTGATG | TTCAGGGTTGATTGTGCGTC |

Additional file 2

| | | | | |
|---|---|---|---|---|
| wy0aba13yf19fm1.1.a.cg.2 | Interferon-induced protein 44 (IFI44L) [*Salmo salar*: NP_001133872] | 1e-23 (35%) | AAGATCCAACGATGAAAGAC | TTGTCGACATCACTACAAAC |
| cdn21p0002p12.f.1.a.cg.2 | Unknown gene product | - | TGCATTGCGTTGATGGATGG | GGAACATCCAAATCACTCCG |
| wy0aba43yo05fm1.1.a.cg.2 | Unknown gene product | - | ATGTCCATGATCGCATAGAAC | AACGCAATCGGTGTTGCTGC |
| wy0aaa36yl22fm1.1.a.cg.2 | Intersectin 1 (SH3 domain protein) [*Danio rerio*: CAX13956] | 2e-82 (60%) | AAGTGATCCGTACTGTGAGG | GGTCCTTGATTGTGAACTGC |
| cdn21p0001n04.f.1.a.cg.2 | Unknown gene product | - | ATCATCATGCCGTCCATGAG | GCTCTAATGCCACAGAGTAC |
| wy0aaa10yo08fm1.1.a.cg.2 | Unknown gene product | - | CTTGACCTTGCAGTCATTGG | TCATCATGCCACTCTAGACC |
| wy0aba24yg16fm1.1.a.cg.2 | ATP-binding cassette transporter [*Aedes aegypti*: EAT35658] | 2e-43 (59%) | TGACCAGTTATGGATGGCAC | CACCAGTATGCTGTTCTGTC |
| wy0aaa30yo05fm1.1.a.cg.2 | Tenascin C [*Gallus gallus*: NP_990787] | 2e-05 (30%) | AGATCAGGTGGCCATGAATG | ATCACAGTTGCCCAGACATC |
| cdn20p0002d19.f.1.a.cg.2 | Unknown gene product | - | CACCTTTCTCATCGTCATCC | TGCAGCTGTCACAAAGCATG |
| oypm10b03e07r1_m13rev.1.a.cg.2 | Unknown gene product | - | AGAAGGGTGTGGCATCTATG | GTCTGATGGACCTGATTTGC |
| cdn21p0004d22.f.1.a.cg.2 | 60S ribosomal protein rpl27 [*Lineus viridis*: ABZ04238] | 2e-52 (85) | GTAAAGCAAGGAGGGATGTC | CATGATTTGTCTGACCTGCG |
| cdn37p0015g12_f.1.a.cg.2 | Unknown gene product | - | TCGTCAGGTGATATTTGTACC | AACTTCATTGGGAGGCATGC |
| oypg09b06c09r1_m13rev.1.a.cg.2 | Unknown gene product | - | AGATGACCTGACATACATTGC | ATCTGGTTGTGACGTAGTGC |
| BQ426799.1.a.cg.2 | Proteasome subunit beta type [*Pheronema raphanus*: ADL27419] | 7e-95 (67%) | GCTGCAGATTGTGCTTACTG | CAGCAACTGATATCCTCTCC |
| wy0aba23ye11fm1.1.a.cg.2 | Unknown gene product | - | TTTCTATAACCAAGACAGGCA | GAGTGTTCCAAGTTGAAGTC |
| wy0aaa2yi18fm1.1.a.cg.2 | Kinesin-like protein KIF9 [*Rattus norvegicus*: NP_001178929] | 2e-21 (43%) | TGGTCATGGAATGAGACCTG | TGTTCATCAGGAGCTCCATC |
| cdn21p0004f02.f.1.a.cg.2 | Unknown gene product | - | CCAGATACAGCGAAGACATG | GATGTCACACTGTAGGTCTC |
| oygd09b07a21r1_m13rev.1.a.cg.2 | Unknown gene product | - | TTCTTGGTTGATAGCAAACTG | GAGTGCTCATGAATGTTGTC |
| wy0aba11ye21fm1.1.a.cg.2 | Multiple epidermal growth factor-like domains 6 [*Loa loa*: EFO21018] | 1e-05 (39%) | AGCCATGCTGCCAGAATGTG | ATCGCTGTACTCTGAGTGTG |
| wy0aaa22yi02fm1.1.a.cg.2 | Unknown gene product | - | ATAGGCCCTTGATTCCACTG | ATACACACGGCATGGTCTAG |
| wy0aaa21yp08fm1.1.a.cg.2 | Unknown gene product | - | TCATTCGATTGGCGAAGGAG | TATGCAGTCTGACTCAGGAC |
| cdn37p0033c18_f.1.a.cg.2 | Unknown gene product | - | TGGATATTGAAGTGCCATCC | GCATGATAATCTTGCTAGCAC |
| oygd10b09m02r1_m13rev.1.a.cg.2 | Unknown gene product | - | TTGGATGGCGTCTCAAAAGC | AGCCGTGTTGATTGATGAGC |
| wy0aaa23yg09fm1.1.a.cg.2 | Unknown gene product | - | TACAGCTGCGCTGAGTTATG | GCTTGGATTGCAGGTCATTG |
| wy0aba18yd01fm1.1.a.cg.2 | Unknown gene product | - | GGCTGTGCACTTGCACATTC | CCAGGGTATAGCATAAGCTC |
| cdn37p0015o06_f.1.a.cg.2 | Zinc finger HIT domain-containing protein 3 [*Danio rerio*: NP_956567] | 2e-22 (42%) | CATTTCAGTGTTTGCGGGAG | TGATGCCAGCTTTCACTTGG |
| cdn19p0002p03.f.1.a.cg.2 | *Crassostrea gigas* big defensin 1 (*Cg*-BigDef1) [*Crassostrea gigas*: AEE92768] | 0 (100%) | TTCGCCTGCTTCCATACTGG | GTCATGGTCACTCCTTATTC |
| cdn21p0002g12.f.1.a.cg.2 | F-box only protein 37 (FBXO37) [*Dicentrarchus labrax*: CBN81533] | 9e-41 (38%) | GAAGAGGAGCTGACATTTC | TCACAGTCCTACTGATGCT |
| cdn20p0004b10.f.1.a.cg.2 | Unknown gene product | - | AAGTCGTATAGGAGCACAGG | GGCTGAGAACATAATCCTCC |

Additional file 2

| | | | | |
|---|---|---|---|---|
| BQ427036.1.a.cg.2 | Unknown gene product | - | AGCTCTCAGAGGAACCGAAG | ACCGGACAACCTTTCATTCC |
| cdn37p0016g17_f.1.a.cg.2 | Unknown gene product | - | ACGTCACTTGTCCATTGTGC | GTTCACGACTGGAAGACATC |
| wy0aaa5ya13fm1.1.a.cg.2 | Baculoviral IAP repeat-containing protein 3 (cIAP2 or BIRC3) [*Rattus norvegicus*: NP_076477] | 3e-10 (53%) | GGGCATCTCATCTATTGCTC | AATCACCATCTTCACCTCCG |
| wy0aaa33ym17fm1.1.a.cg.2 | Unknown gene product | - | GAGGAATTACGTGGACATGC | TGGAGACTTCCTGTTGTGAC |
| wy0aaa40yd07fm1.1.a.cg.2 | Phosphoserine aminotransferase 1 isoform 1 [*Haliotis discus discus*: ABO26670] | 2e-78 (66%) | TCCAAACTCAAGTCCCAAGC | GCGTTGTATAGAGAAGCTCG |
| cdn20p0001l08.f.1.a.cg.2 | Glutamine synthetase (Glutamine-tRNA synthetase) [*Crassostrea gigas*: CAD90162] | 0 (100%) | GTCCTGTCTGGAACTATGAC | TGAAGACCTCACACATCACC |
| wy0aaa8ye17fm1.1.a.cg.2 | Unknown gene product | - | ACAAAGTGAGGCTGAACTCC | TGACATCGTATCCTGGCATG |
| cdn37p0024j10_f.1.a.cg.2 | Unknown gene product | - | ATCCGGTTAGACTGTGATCG | TAGGACCCATCACTGAACTG |
| wy0aba38yf11fm1.1.a.cg.2 | Unknown gene product | - | ACAACTCTCTGGTTCTGTGG | TCCAAACAAGGGACGATCTG |
| wy0aaa20yo07fm1.1.a.cg.2 | Ribosome biogenesis protein BOP1 [*Salmo salar*: NP_001167382] | 8e-100 (75%) | AGGAATGGTTCAGTGTGTCC | GTTTCTTGGACAGCTCTTGC |
| wy0aba38yp06fm1.1.a.cg.2 | Unknown gene product | - | ATTGTGCACTCAGAAGAGGC | AACTCGGCTCTCTGTAATGG |
| cdn37p0014c17_f.1.a.cg.2 | Glutathione S-transferase zeta [*Kryptolebias marmoratus*: ABW88890] | 2e-65 (56%) | GCAGAATGGGCTAAGTTCTG | AGAGTCTAGCTGCTGAACAC |
| cdn19p0003j04.f.1.a.cg.2 | Unknown gene product | - | GTGTTGACTCGATACAGCAG | ATGGTGGAAAGATGGCTTCG |
| cdn37p0006p09.f.1.a.cg.2 | Kelch repeat-containing protein [*Capsaspora owczarzaki*: EFW39768] | 2e-12 (40%) | TACCGTACTACCACGAGAAC | GTACGCTAGCACTGATGAGA |
| cdn37p0004m04.f.1.a.cg.2 | Somatostatin receptor [*Hydra magnipapillata*: XP_002160368] | 2e-17 (35%) | CTGTAACAGATGGCGTCATG | AGATTGAACTGGTCACCCAG |
| wy0aaa20yi09fm1.1.a.cg.2 | C-type lectin 2 like protein [*Crassostrea gigas*: CAE18170] | 0 (100%) | GTCATCTGACCACAATTACAG | TCGATAGCAGCATTCCAGAG |
| oygd09b07p20r1_m13rev.1.a.cg.2 | Mitochondrial malate dehydrogenase precursor [*Nucella lapillus*: AAF27650] | 7e-122 (67%) | TGGCTGCAGATCTGAGTCAC | TCATCTCTGGTCATTCCTGG |
| cdn37p0017g17_f.1.a.cg.2 | Unknown gene product | - | GTGTTCCACATGCAGGAAGC | TCGATGCTGGCATTGTTGTC |
| cdn37p0023e09_f.1.a.cg.2 | Zinc finger protein [*Ciona intestinalis*: FAA00184] | 6e-09 (39%) | AAGAATTGGAGGCTCAGGAG | ATTGCTCATGCTGCTACACC |
| cdn37p0014n20_f.1.a.cg.2 | Unknown gene product | - | ATTTGCTCTACTTGTTGCTGC | AACATGCACATCCAGAGTCG |
| cdn37p0012j23_f.1.a.cg.2 | Unknown gene product | - | TCGAGTGTAACTCGAGAGAC | TTCTACATCTTGCGCTGGCG |
| wy0aba36yg16fm1.1.a.cg.2 | Unknown gene product | - | GCAGTTTACACCCATTGACC | TTGGATGAGGCTATCTCATAC |
| wy0aaa11yg08fm1.1.a.cg.2 | Unknown gene product | | TAGTCACAAATGCGGCAACG | ATCTATAGTGATCGGCACGG |
| wy0aba34yj17fm1.1.a.cg.2 | Keratinocyte-associated protein 2 [*Rattus norvegicus*: NP_001099914] | 5e-21 (59%) | GTCCATCGAGTATGCGTAAC | GTTGCTTTGGTTGGAACAGC |
| cdn19p0002a02.f.1.a.cg.2 | Baculoviral IAP repeat-containing protein 4 (API3 or BIRC4) [*Xenopus laevis*: NP_001089083] | 8e-22 (29%) | AGACAGCAACGTTAATACAC | GTACATAGAGGCTTGATAGC |
| wy0aba13yd03fm1.1.a.cg.2 | Unknown gene product | - | ATTCCAGGACGCACAGATAC | TTCCTTCTCACAGCTGTCTG |
| cdn37p0008f02_f.1.a.cg.2 | Trafficking protein particle complex 6b [*Danio rerio*: AAH83391] | 3e-58 (68%) | CCATCAGGGAGTGTATGTTC | GACTTCCGCTGTTACAACAC |

Additional file 2

| | | | | |
|---|---|---|---|---|
| cdn20p0005l03.f.1.a.cg.2 | Unknown gene product | - | TATGCCGTGTTGTGTCAGAC | ATCGCTGTCCACAGAACTAC |
| cdn20p0003l17.f.1.a.cg.2 | Abhydrolase domain containing 4 [*Xenopus tropicalis*: NP_001017287] | 1e-34 (53%) | AAGTACCTCCGGAATGAGAG | ACTGCTTCGCATATCCTCAG |
| cdn19p0002h24.f.1.a.cg.2 | Unknown gene product | - | TTCAAACGAGCCTACTTTCAC | GAAACTGCAAGACGTTCTGG |
| wy0aba34yh24fm1.1.a.cg.2 | Unknown gene product | - | AACAAAGATGGCGGACTACG | AATGCACACCTTGTGTTCCG |
| cdn20p0001f03.f.1.a.cg.2 | Hexokinase [*Crassostrea gigas*: CAJ28915] | 0 (100%) | ATCCTCAAGATGCCTTCCAC | TCTTCATCTCCTCGTGCAGG |
| wy0aba9yd11fm1.1.a.cg.2 | Poly(U)-specific endoribonuclease-D [*Xenopus laevis*: NP_001104223] | 9e-12 (47%) | GCCAGATTTACCTGGAATGG | ATGCGGTGTAGATAGCGATG |
| oyge10b14d14r1_m13rev.1.a.cg.2 | Retinoblastoma-binding protein 4 (rbbp4) [*Aedes aegypti*: XP_001654079] | 0 (92%) | TGCTTCACATTACGACAGCG | TGTCTTCGTGGCGATGATAC |
| cdn37p0034j21_f.1.a.cg.2 | Mediator of RNA polymerase II transcription subunit 7 [*Camponotus floridanus*: EFN70039] | 4e-71 (58%) | AGACTCTCCGTGTCATGATG | TAGTTGGTCACACGGTTCAG |
| cdn37p0002j17.f.1.a.cg.2 | Unknown gene product | - | CCAGTCTCCTTAAGAACCAG | TGCAACCTACATGTAACTTGG |
| cdn37p0002d10.f.1.a.cg.2 | *Crassostrea gigas* MyD88 adaptor (*Cg*-Myd88) [*Crassostrea gigas*: ABF81403] | 0 (100%) | AGGTACCGGCTGTGATACGA | TTCAAACGCCACCAAGACTG |
| EU073062.1.a.cg.2 | 5-aminolevulinate synthase [*Crassostrea gigas*: ABU50033] | 0 (100%) | AACACGACATCTACGTCCAG | TTCCAGACCATTGTCACTCC |
| BQ426622.1.a.cg.2 | Cathepsin L-like cysteine proteinase [*Haliotis diversicolor supertexta*: ACR43934] | 3e-95 (68%) | TCCTGTTGGTCCTTCAGCAC | GCATTGTCCATAAGACCACC |
| cdn19p0003b02.f.1.a.cg.2 | Carbonyl reductase 1 (NADPH) [*Cricetulus griseus*: BAB62840] | 9e-85 (57%) | AAGTTGGGATCAGTGTCCTC | TCGGACCTTTGTGGGATGAC |
| cdn21p0001k16.f.1.a.cg.2 | Peroxiredoxin [*Pinctada fucata*: ADC35419] | 4e-92 (86%) | GATCACCATGAATGACCTCC | ACATTGGGACGGATTGTGTC |
| cdn21p0003k02.f.1.a.cg.2 | Proliferation-associated protein 2G4 [*Camponotus floridanus*: EFN63603] | 6e-108 (65%) | GCACACACACTTGTAGTTGG | TGGTTTGACCAATCGGAGTG |
| cdn37p0017n22_f.1.a.cg.2 | Bhlhzip transcription factor max/bigmax [*Aedes aegypti*: XP_001662341] | 2e-36 (79%) | GGAGAAATCAGTCATGGACG | CTAGTACAGATGCACTGCTG |
| cdn37p0022h15_f.1.a.cg.2 | Vacuolar-sorting protein SNF8 [*Ascaris suum*: ADY44444] | 2e-66 (59%) | AGTTAACCATGGACCACACC | CATGTAATCCAAGGCTCTGG |
| cdn37p0026g18_f.1.a.cg.2 | Sulfotransferase 1B [*Gallus gallus*: CAD41949] | 1e-27 (52%) | CCACGATGTCTATTCCATCG | TTCTGGGCATACTCTGCGTC |
| cdn37p0027n21_f.1.a.cg.2 | Etoposide induced 2.4 mRNA [*Gallus gallus*: NP_001006316] | 1e-30 (41%) | GCTTGTTATGGTTCACCGAG | CAGAGAGCTTATCCACAGCC |
| oygd09b07i24r1_m13rev.1.a.cg.2 | Tumor necrosis factor ligand superfamily member 10 (TNFSF10) [*Sus scrofa*: NP_001019867] | 1e-09 (26%) | GGATACGCAAGAGGAACTGC | TGGACATTAACGACACGCGC |
| oygd10b09i21r1_m13rev.1.a.cg.2 | UDP-N-acetylglucosamine--peptide N-acetylglucosaminyltransferase 110 kDa subunit [*Camponotus floridanus*: EFN64211] | 1e-103 (65%) | TGGACACTTCCAGACACAGG | AGCACTATCATAACGGCTCC |
| oyge10b13e16r1_m13rev.1.a.cg.2 | Nucleoside diphosphate kinase B [*Haliotis discus discus*: ABO26651] | 5e-69 (76%) | CAGTTGTTGCAATGGTCTGG | ACAGTAATCTCCCCTGATGG |
| oyio09b02o09r1_m13r.1.a.cg.2 | Mitochondrial methylmalonate-semialdehyde dehydrogenase [*Perca flavescens*: ADX97168] | 5e-74 (76%) | TTGGACCAGTTCTGGTGAGC | GCCATTGGTCGTGAAGATGG |
| oypg10b07n22r1_m13rev.1.a.cg.2 | Dual specificity protein phosphatase 7 [*Dicentrarchus labrax*: CBN80861] | 3e-15 (77%) | AACAATACGCAGGGACATCC | TCACGTTTGCCGAACACTGC |
| wy0aaa15yc10fm1.1.a.cg.2 | Beta-1,3-galactosyltransferase 1 [*Homo sapiens*: NP_066191] | 3e-25 (37%) | GGCCTAATGAATGCAAGTCG | ACCTGTGTACGGACTCATCC |
| wy0aaa17ym11fm1.1.a.cg.2 | Caspase 7, apoptosis-related cysteine peptidase [*Xenopus laevis*: NP_001091272] | 2e-39 (50%) | ATCACCAGGAAGGATCATGG | GTTCATCCGAACACGACTCG |

Additional file 2

| | | | | |
|---|---|---|---|---|
| wy0aaa24yn02fm1.1.a.cg.2 | Glucan endo-1,6-beta-glucosidase [*Paenibacillus curdlanolyticus*: EFM10096] | 1e-46 (49%) | CATGTACTGGAACATCGCAC | GGTATTCTTCGTCGTATCGC |
| wy0aaa26yg07fm1.1.a.cg.2 | Cell division cycle 27 homolog (*S. cerevisiae*), isoform CRA_a [*Rattus norvegicus*: EDM06315] | 2e-77 (71%) | ATGCCACAGATGAAGACGAG | ACTTTCATCGCTCTCTGTGG |
| wy0aaa30yd03fm1.1.a.cg.2 | Ornithine aminotransferase [*Aedes aegypti*: EAT43242] | 7e-119 (73%) | AAGTACGGGAACTGTGTACC | CACTTCTTGTGGTCTACAGC |
| wy0aaa30yg14fm1.1.a.cg.2 | Leucine aminopeptidase 2 [*Clonorchis sinensis*: ACR2708] | 7e-84 (63%) | TCCCATCTGGACATTGCTGG | CCAACACACTTAACCATGCG |
| wy0aaa35yf07fm1.1.a.cg.2 | Vacuolar protein sorting-associated protein 28-like protein [*Harpegnathos saltator*: EFN81106] | 4e-79 (73%) | TCAACCGCATACTGCACGAC | AACTGACCGTACAGATGTGG |
| wy0aaa4yf11fm1.1.a.cg.2 | Tumor protein p53 inducible protein 3 (TP53I3) [*Xenopus laevis*: NP_001085954] | 1e-60 (51%) | CCTGATATTGGACTGTGTGG | GTATCTATCGTCCTTCCACC |
| wy0aba14yi09fm1.1.a.cg.2 | Glutathione oxidoreductase [*Candidatus Vesicomyosocius okutanii*: BAF61405] | 1e-08 (85%) | AGGCCCAGTTTGACGAGACG | CGGAGTGTCACCAACTCCTC |
| wy0aba15yj02fm1.1.a.cg.2 | Toll9 [*Anopheles gambiae*: AAL37903] | 1e-15 (33%) | TTCAATCTGGCGACACCGAG | GGACAGAGAATTACGCAGAC |
| wy0aba28yj20fm1.1.a.cg.2 | Saposin [*Aedes aegypti*: EAT45724] | 7e-14 (51%) | GCAGCATTGCTTGGATACAG | CAGAGATCACATGATGCTGG |
| AJ544883.2.a.cg.2 | *Crassostrea gigas* growth/differentiation factor 2 (GDF2) precursor [*Crassostrea gigas*: CAD67714] | 0 (100%) | GCATGAGAGGAACTTATCGC | TGAATTGCCAACCTTGGACG |
| BQ426894.1.a.cg.2 | Beta-hydroxysteroid dehydrogenase type 11 [*Haliotis diversicolor supertexta*: ADV02385] | 8e-89 (56%) | AAGATGGCATCCACACTACC | TTCTTCGTTGGTTAGCGTCC |
| cdn20p0003l18.f.1.a.cg.2 | Apoptosis regulator protein, Bcl-2 family [*Trichinella spiralis*: EFV58758] | 4e-13 (35) | AGGATAGCACTCTATGCAGG | TCAACTCCTAGCAACCATGG |
| cdn20p0005a13.f.1.a.cg.2 | Peptide methionine sulfoxide reductase [*Salmo salar*: ACI67235] | 2e-86 (71%) | CTGGATGATAAAGGACTCGG | AATACTGCTGGTGGTAGTCC |
| cdn21p0002a18.f.1.a.cg.2 | Fructose 1,6-bisphosphatase [*Marsupenaeus japonicus*: BAJ23881] | 4e-126 (70%) | TCACCTGTCTACTGGTATCG | AAATGGAGCCAATGGACACC |
| cdn37p0005n23.f.1.a.cg.2 | TGF beta-activated protein kinase kinase kinase 7 [*Salmo salar*: ACN10470] | 4e-24 (52%) | GTTCAAACAACTGAGGGAGG | TCGAGTAACGTAGTCGTTGG |
| cdn37p0006e18.f.1.a.cg.2 | Hormone-sensitive lipase [*Sus scrofa*: AAT95416] | 2e-27 (69%) | TTGTCACCTTGACCCCTTGC | CTTGCCGTGGAGAAGTTCAG |
| cdn37p0012m10_f.1.a.cg.2 | Trans-2,3-enoyl-CoA reductase [*Xenopus laevis*: NP_001085733] | 4e-28 (48%) | GGCAGTCTAGGATTATCAGG | CAAAGGACCTGCATACTCTG |
| cdn37p0031k04_f.1.a.cg.2 | Mitochondrial glutamyl-tRNA synthetase [*Glossina morsitans morsitans*: ADD18343] | 2e-35 (40%) | GAACATGTCCTCTCATTCGG | TTATGAGGTCAGGGTCATCG |
| cdn37p0034c08_f.1.a.cg.2 | DNA ligase I [*Xenopus laevis*: AAB37754] | 3e-151 (75%) | TTCCTCTGTAGAACCAGACC | TCTGGATCAACGATTCCTGC |
| oyge09b12f10r1_m13rev.1.a.cg.2 | Amylo-1,6-glucosidase (Glycogen debranching enzyme) [*Camponotus floridanus*: EFN70541] | 9e-106 (58%) | GGAGGTGTTGTATGATCTGG | CAGTTTGTTGAGAGAGGTCC |
| oyge10b13f12r1_m13rev.1.a.cg.2 | Guanine nucleotide binding protein (G protein), gamma 7 [*Xenopus tropicalis*: NP_001017133] | 4e-06 (43%) | CAACATGCTCAGGATGATGC | CATCGCCCAAAGTTGCTCAG |
| oypg09b06c05r1_m13rev.1.a.cg.2 | IQ motif and SEC7 domain-containing protein 1 [*Camponotus floridanus*: EFN63168] | 8e-36 (50%) | TTGTAGAGGACCTTCGAGAG | TCTTCGATTCGCAGTGTCTC |
| oypg09b06n02r1_m13rev.1.a.cg.2 | Glutaryl-Coenzyme A dehydrogenase [*Xenopus tropicalis*: AAI35459] | 2e-53 (88%) | GAGTTGCACGAGATATGCTC | AGGCATGGATGTCGTGTGTG |

Additional file 2

| | | | | |
|---|---|---|---|---|
| oypm10b04d07r1_m13rev.1.a.cg.2 | 26S proteasome non-ATPase regulatory subunit 8 [*Ictalurus punctatus*: ADO29154] | 2e-88 (66%) | TGGCTGAACAGACCATAGAG | CTCCATTAGAGTACGAGGTC |
| wy0aaa18yg19fm1.1.a.cg.2 | Iron/zinc purple acid phosphatase-like protein precursor [*Danio rerio*: NP_001092720] | 3e-15 (67%) | GCAGGTTTCTGATGACAAGG | CATCTTGTCCTTGTTGCGAC |
| wy0aaa18yn10fm1.1.a.cg.2 | Glutamyl-tRNA synthetase, cytoplasmic [*Ixodes scapularis*: EEC07187] | 4e-83 (60%) | CAGCTACATGATTGTCTCCC | CCTTCTTGATCTTGTCCTCG |
| wy0aaa1yi08fm1.1.a.cg.2 | E3 ubiquitin-protein ligase HUWE1 [*Salmo salar*: ACN58725] | 4e-83 (85%) | AAAGAAGGTGCTTGTCAGGC | TACATGGCATTAGTGGCAGG |
| wy0aaa24ym23fm1.1.a.cg.2 | 6-phosphogluconolactonase [*Esox lucius*: ACO13366] | 2e-13 (55%) | CCACCCAAGGAGAACTGATC | CACTTAGATGTTGGACTGGG |
| wy0aaa2yi20fm1.1.a.cg.2 | Folylpolyglutamate synthase [*Xenopus tropicalis*: NP_001096413] | 2e-26 (36%) | GCTTATAGAACCACTGGTGG | CCTTCTCATGCTTCTGTTGC |
| wy0aaa36yc16fm1.1.a.cg.2 | Profilin-4 [*Mus musculus*: NP_082652] | 2e-27 (48%) | AAGGTGCTGAAGTCTTGGAC | GTGATGTACATGGTCTGTCG |
| wy0aaa37yf05fm1.1.a.cg.2 | Delta-6 fatty acid desaturase [*Rattus norvegicus*: BAA75496] | 8e-112 (48%) | TGTACTGTTAGCCACCTCAC | TTCCACCAGTGAGATGATGC |
| wy0aba18yn10fm1.1.a.cg.2 | D-glucuronyl C5-epimerase [*Harpegnathos saltator*: EFN87460] | 1e-19 (64%) | ACAGTTGCTGAGTGAAGTGG | TCTAATTGTGGGGCGACAGC |
| wy0aba22yf07fm1.1.a.cg.2 | Integrin alpha-4 [*Mus musculus*: NP_034706] | 9e-33 (37%) | TTGGTGCATACAGATCCGAC | TTCGTCCATCACTACATGGC |
| wy0aba34ye13fm1.1.a.cg.2 | Beta 1,4-N-acetylgalactosaminyltransferase [*Trichoplusia ni*: AAT11926] | 2e-48 (41%) | CACCATACACCCACAAGATG | GCATTCCCTTGTTGAAAGGC |
| wy0aba42ya22fm1.1.a.cg.2 | Methionine-R-sulfoxide reductase B3, mitochondrial [*Gallus gallus*: NP_001186507] | 1e-48 (54%) | CTGTAACTCGCACATAGGTC | TGTAGGTAGCAGCAATGACC |
| wy0aba43yi02fm1.1.a.cg.2 | Endoglucanase [*Mizuhopecten yessoensis*: BAH85844] | 5e-37 (64%) | ACAACTTCCGGACTAACGAG | ATCTAGCATGCAGGTGTTGG |
| AB179775.1.a.cg.2 | *Crassostrea gigas* lysozyme (CGL-1) [*Crassostrea gigas*: BAD19059] | 0 (100%) | CACCAAGTAATCATGCAGCG | CACGAGTATGAGTACACGTC |
| oyge10b13d08r1_m13rev.1.a.cg.2 | *Crassostrea gigas* big defensin 3 (*Cg*-BigDef3) [*Crassostrea gigas*: AEE92778] | 0 (100%) | AGAAGAAGGTGAGACGAG | TGATCCGCACACACCAAACG |
| oypg10b08d05r1_m13rev.1.a.cg.2 | *Crassostrea gigas* extracellular superoxide dismutase 1 (*Cg*-EcSOD1) [*Crassostrea gigas*: AAY60161] | 0 (100%) | AGAGGTGAATGCTACCAGG | AGGCCAAGAATTCCGTCTG |
| oygd09b07k07r1_m13rev.1.a.cg.2 | Integrin beta-PS [*Camponotus floridanus*: EFN70798] | 1e-15 (35%) | CCCACCTAGTGCCAGTCAAG | GAACTTTGACTTGTGTGACGT |
| oypg10b08d05r1_m13rev.1.a.cg.2 | Superoxide dismutase-containing domain [*Crassostrea gigas*: oypg10b08d05r1_m13rev.1.a.cg.2] | 0 (100%) | AAGTTGTCGACCCGAACACG | ACGCTAATAACTGGCTCTGG |
| cdn37p0004a18.f.1.a.cg.2 | Alpha2-macroglobulin [*Chlamys farreri*: AAR39412] | 1e-79 (45%) | TGTGGGACAAAGGAAGAGTTG | ACAAACAGGGCAGGACTTGA |
| wy0aba12yp08fm1.1.a.cg.2 | *Crassostrea gigas* Cystatin B-like protein [*Crassostrea gigas*: ADI33157] | 4e-34 (74%) | GAGATTCCCCCTCACTCCTC | TGCTGAAAGCCTCCAAATCT |
| wy0aba28yc21fm1.1.a.cg.2 | Cathepsin B sequence [*Pinctada fucata*: ADX32985] | 3e-53 (71%) | TTGATTCCCGCACACAGTGG | CTTCAACAGCTCCGAAAGCC |
| cdn19p0002p03.f.1.a.cg.2 | *Crassostrea gigas* big defensin 2 (*Cg*-BigDef-2) [*Crassostrea gigas*: AEE92775] | 0 (100%) | GGAGAGAAAATTCTGACCATGAC | CATAGTTTATCCCCTCCGTC |
| oygd09b07e15r1_m13rev.1.a.cg.2 | *Crassostrea gigas* lipopolysaccharide-induced TNF-alpha factor (*Cg*-LITAF) [*Crassostrea gigas*: ABO70331] | 0 (100%) | CCTACCAGGGCCAGACAAG | GCGGCCGAGCACCTGTCG |
| cdn21p0002f21.f.1.a.cg.2 | *Crassostrea gigas* Drac3-like protein [*Crassostrea gigas*: ACB29737] | 0 (100%) | GATAATAGTGCGACGGAGTG | CATCAGCATACAGGTCTTCC |
| cdn37p0003g06.f.1.a.cg.2 | *Crassostrea gigas* transcription factor Tal (*Cg*-tal) [*Crassostrea gigas*: AAK72692] | 0 (100%) | CCAAAAGACTCTGGTTACATTTGCC | GACGTTCTGTTGCCGCCAGCG |

Additional file 2

| | | | | |
|---|---|---|---|---|
| GenBank: EF190193 | *Crassostrea gigas* interleukin 17 (*Cg*-IL17) [*Crassostrea gigas*: ABO93467] | 0 (100%) | ACTGAGGCTCGATGCAAGTG | AGCCTTCTTGCTTCATGTGG |
| cdn20p0003h10.f.1.a.cg.2 | *Crassostrea gigas* activin type II receptor [*Crassostrea gigas*: CAR92545] | 0 (100%) | CACTGGAGGACATGCAAGAA | GGCTGCAACCTGCTCTAAAC |
| cdn37p0018j18_f.1.a.cg.2 | Inhibitor of apoptosis [*Aplysia californica*: ADI24344] | 2e-25 (36%) | CATTATGGAAGCAGATAGATC | ATGATGTCATCTTCCTTTGTC |
| oygd09b07l03r1_m13rev.1.a.cg.2 | *Crassostrea gigas* calreticulin (CRT) [*Crassostrea gigas*: BAF63639] | 0 (100%) | ACTGGGATGACGAGATGGAC | GCCAAAGATCAAATCCAACG |
| cdn37p0016f07_f.1.a.cg.2 | Heat shock protein 22 isoform 1 (HSP22) [*Venerupis philippinarum*: ACU83231] | 3e-22 (61%) | GGCAAAGACCCATTTGGTAA | ACAGTCAAGTTCCGGTCCAC |
| oypg09b05c22r1_m13rev.1.a.cg.2 | *Crassostrea gigas* heat shock protein 68 kDa (HSP68) [*Crassostrea gigas*: BAD15285] | 0 (100%) | GCATGTGAGCGAGCAAAACG | TGGCAGCTTGAACAGCAGC |
| cdn37p0013p20_f.1.a.cg.2 | 26S proteasome non-ATPase regulatory subunit 7 (PSMD7) [*Harpegnathos saltator*: EFN75539] | 5e-100 (81%) | GCGAATTGTGGGATGGTATC | TCATGCACTTCTTCCACTGC |
| cdn37p0012a07_f.1.a.cg.2 | Rac GTPase-activating protein 1 [*Camponotus floridanus*: EFN67432] | 6e13 (44%) | GTTAAGTCATCCCTACCATTC | ACATGGTGGATACGCAGAAG |
| cdn19p0004c06.f.1.a.cg.2 | Galectin [*Crassostrea virginica*: ABG75998] | 2e-51 (50%) | ACGAAACGCTCTGATTGGTG | TTAGTGGCATGGTAGGTCTG |
| wy0aba16yo20fm1.1.a.cg.2 | Rhamnospondin 1, L-rhamnose-binding lectin [*Hydractinia symbiolongicarpus*: ABD95939] | 7e-17 (44%) | AGATGATTGTGAAAGCAGCGA | ACTGTAGCGGTCATGCTCTG |
| wy0aba23yg22fm1.1.a.cg.2 | *Crassostrea gigas* proline rich protein (*Cg*-PRPL) [*Crassostrea gigas*: ACQ72941] | 0 (100%) | CACCATGTTCTCTCGGAGGA | ATCTGCAATGTCAACCCTCTG |
| wy0aba36yl08fm1.1.a.cg.2 | *Crassostrea gigas* proline rich protein (*Cg*-PRPC) [*Crassostrea gigas*: ACQ72971] | 0 (100%) | CACCATGTTCTCTCGGAGGA | GTCTGCAATGTTAACCCTCAG |
| AJ582629.1.a.cg.2 | *Crassostrea gigas* hemocyte defensin (*Cg*-Defh) [*Crassostrea gigas*: ABD66302] | 0 (100%) | GTTGTAGAGCGGGCTACTGTG | CTTGGTCAGATTCAGACTGG |
| oypm09b01c03r1_m13rev.1.a.cg.2 | MAP kinase-interacting serine/threonine-protein kinase 1 (MKNK1) [*Mus musculus*: NP_067436] | 5e-116 (61%) | ACATGCTAGAGGCAAGACAC | CACATGGAAATCCTGTTGCC |
| wy0aba16yo20fm1.1.a.cg.2 | Rhamnospondin 1, L-rhamnose-binding lectin [*Hydractinia symbiolongicarpus*: ABD95939] | 7e-17 (44%) | TGAAGTTGGAGAGTCATGGG | ACTCTGCCATGACCACAATG |
| wy0aaa40yb12fm1.1.a.cg.2 | Neural-cadherin [*Camponotus floridanus*: EFN64630] | 2e-14 (45%) | GCTAGCGTTCAGTCAACTCG | ACCTCATGTCTGGTACACAG |
| cdn37p0026i19_f.1.a.cg.2 | *Crassostrea gigas* tyrosine phosphatase [*Crassostrea gigas*: ACH42087] | 0 (100%) | CGAAATCGTCTTACGAACGC | GTTAGCAAGATCCCGTTGAG |
| oyge10b14n20r1_m13rev.1.a.cg.2 | RAB18, member RAS oncogene family [*Xenopus tropicalis*: NP_001017281] | 3e-31 (71%) | GTCAGGGATACTGTGCTGTC | CAGTCCTGGAGTTCACTCTG |
| wy0aaa8yo10fm1.1.a.cg.2 | AP-2 complex subunit beta-1, putative [*Ixodes scapularis*: XP_002412040] | 5e-28 (70%) | GCAATGGATGTATACCAAGGC | CGTTACGGAATTAGGTCCAC |
| oyge09b12a09r1_m13rev.1.a.cg.2 | Cyclin G1 [*Xenopus laevis*: NP_001090435] | 1e-21 (33%) | GTTGGAAGTGGTAGTCAGTG | CTCCAAACTACATGTGGCAC |
| wy0aba43ye24fm1.1.a.cg.2 | Methyltransferase-like protein [*Latrodectus hesperus*: ADV40120] | 6e-20 (40%) | TCCTTCGCAACTTCGTCTTC | TATCGTACCCAGCACGTATC |
| cdn37p0005n23.f.1.a.cg.2 | TGF beta-activated protein kinase kinase kinase 7 (MAP3K7) [*Salmo salar*: ACN10470] | 4e-24 (52%) | CGGAGGAGTTCAAACAACTG | TCGAGTAACGTAGTCGTTGG |
| oyge10b14i13r1_m13rev.1.a.cg.2 | Collagen receptor related protein [*Crassostrea gigas*: AAQ13478] | 0 (100%) | GTTTAGTGACGACTCAGAGC | GTTGGCTTCAGATTCTGCTG |
| cdn37p0030b11_f.1.a.cg.2 | Calcium and integrin-binding protein 1 [*Camponotus floridanus*: EFN62878] | 8e-50 (64%) | AGGAGATGGAGCAACTCATC | ATTCTCCATGTCTACAGCCG |
| cdn21p0001h19.f.1.a.cg.2 | Small GTPase Cdc42 [*Schizophyllum commune*: XP_003036258] | 7e-63 (60%) | ACTGGCAAGAGAACTGAAGG | CTTCTTCACAGGTGATCAGC |

Additional file 2

| | | | | |
|---|---|---|---|---|
| cdn37p0017g12_f.1.a.cg.2 | Rho-related GTP-binding protein RhoQ [*Danio rerio*: NP_956112] | 1e-21 (37%) | TCTGGATTCCTAAGATCCGG | GTATCGAATGCTTGCCATGG |
| cdn20p0001i19.f.1.a.cg.2 | Gamma-aminobutyric acid receptor-associated protein-like 2 [*Osmerus mordax*: ACO09769] | 1e-40 (75%) | ATAAGGAAGTCGCTCACCTC | TTCTCTCTGGGTACTTGTCC |
| wy0aba31yf08fm1.1.a.cg.2 | Beclin-1-like protein [*Camponotus floridanus*: EFN67583] | 4e-16 (91%) | GAAGAACAGTGGACCAAAGC | GAATACTGGTCACAACTCCC |
| wy0aaa20yc12fm1.1.a.cg.2 | Early growth response protein 1 (Egr1) [*Ovis aries*: NP_001135978] | 6e-39 (68%) | CTACCTCCACAAGCGACATG | ACGTCGTTACTATGTGAGGG |
| oyge10b13c08r1_m13rev.1.a.cg.2 | Src tyrosine kinase 2 [*Aplysia californica*: ACI23623] | 6e-103 (88%) | GGAGTTCTTCACCATAGAGG | AGTCAACGTCAACAGTGAGC |
| cdn19p0003b05.f.1.a.cg.2 | Transglutaminase (Protein-glutamine gamma-glutamyltransferase K) [*Bos taurus*: DAA25714] | 1e-39 (33%) | AGCAGCATGACGATTGACAG | ATTCCTTCGCTTGCTTCCTG |
| oygd09b08h18r1_m13rev.1.a.cg.2 | *Crassostrea gigas* actin (*Cg*-actin) [*Crassostrea gigas*: ACQ72911] | 0 (100%) | CCATGTACGTCGCCATCCAG | GATCACGTCCAGCGAGATCC |
| wy0aaa16yh01fm1.1.a.cg.2 | *Crassostrea gigas* selenium-dependent glutathione peroxidase [*Crassostrea gigas*: ABS19600] | 0 (100%) | TGCTGTAAACTGACATATGACA | GCCTATCGTTGAGAACCATG |
| wy0aaa31yh04fm1.1.a.cg.2 | *Crassostrea gigas* catalase [*Crassostrea gigas*: ABS18267] | 0 (100%) | CACCCAGAAGTTGATCCGC | GCTCACATTGCTGACGGAG |
| cdn21p0003m03.f.1.a.cg.2 | *Crassostrea gigas* transcription factor Rel 1 (*Cg*Rel1) [*Crassostrea gigas*: AAK72690] | 0 (100%) | GCTGAACCAGAACCTCATGA | CGAAGGACATGTTCTGATCC |
| wy0aaa18yn15fm1.1.a.cg.2 | *Crassostrea gigas* tissue inhibitor of metalloproteinase (*Cg*-TIMP) [*Crassostrea gigas*: AAG42824] | 0 (100%) | TGAGGCAGTACAACTTCCTTCTATT | ACCCCTTGAATATGTCTCTCTTCTT |
| cdn37p0016c10_f.1.a.cg.2 | *Crassostrea gigas* peptidoglycan recognition protein L (*Cg*-PGRP_L1) [*Crassostrea gigas*: BAH66799] | 0 (100%) | GCGTAGCAAGTCGGGAGTCT | CCGTAAGCGTTGTGGTGATCT |
| wy0aba28yp05fm1.1.a.cg.2 | *Crassostrea gigas* bactericidal permeability increasing protein (*Cg*-BPI) [*Crassostrea gigas*: AAN84552] | 0 (100%) | GATAGAAATAGGAATGGACGG | GTTATAGATCCACGCTGCTCC |
| wy0aaa11yi05fm1.1.a.cg.2 | *Crassostrea gigas* toll-like receptor 1 (*Cg*Toll-1) [*Crassostrea gigas*: ADV16385] | 0 (100%) | CTCTAAGCGTTCCATCGTTC | CTCAGTGTTACTAGCAGACG |
| wy0aaa25yf18fm1.1.a.cg.2 | Tumor necrosis factor receptor-associated factor 3 (TRAF3) [*Branchiostoma belcheri*: ABN04152] | 8e-24 (78%) | CAGCCAGCCATTTTACACCAGTC | CCGTTTAAAACTGCTGCTTGTGG |
| wy0aaa1yk06fm1.1.a.cg.2 | *Crassostrea gigas* laccase 1 (multicopper oxidase) [*Crassostrea gigas*: ACH42090] | 0 (100%) | CATCCATGTAAGGGGTCCCCGTCTG | GATGGGTGGGAGGCGAAACGACTTG |
